# Unusual life cycle and impact on microfibril assembly of ADAMTS17, a secreted metalloprotease mutated in genetic eye disease

**DOI:** 10.1038/srep41871

**Published:** 2017-02-08

**Authors:** Dirk Hubmacher, Michael Schneider, Steven J. Berardinelli, Hideyuki Takeuchi, Belinda Willard, Dieter P. Reinhardt, Robert S. Haltiwanger, Suneel S. Apte

**Affiliations:** 1 Department of Biomedical Engineering, Cleveland Clinic Lerner Research Institute, Cleveland Clinic Foundation, Cleveland, OH, 44195, USA; 2 Department of Biochemistry and Cell Biology, 450 Life Sciences Building, Stony Brook University, Stony Brook, NY 11794-5215, USA; 3 Complex Carbohydrate Research Center, University of Georgia, Athens, GA 30602, USA; 4 Mass Spectrometry Laboratory for Protein Sequencing, Lerner Research Institute, Cleveland Clinic, Cleveland, USA; 5 Department of Anatomy and Cell Biology and Faculty of Dentistry, McGill University, Montreal, Quebec, H3A OC7, Canada

## Abstract

Secreted metalloproteases have diverse roles in the formation, remodeling, and the destruction of extracellular matrix. Recessive mutations in the secreted metalloprotease *ADAMTS17* cause ectopia lentis and short stature in humans with Weill-Marchesani-like syndrome and primary open angle glaucoma and ectopia lentis in dogs. Little is known about this protease or its connection to fibrillin microfibrils, whose major component, fibrillin-1, is genetically associated with ectopia lentis and alterations in height. Fibrillin microfibrils form the ocular zonule and are present in the drainage apparatus of the eye. We show that recombinant ADAMTS17 has unique characteristics and an unusual life cycle. It undergoes rapid autocatalytic processing *in trans* after its secretion from cells. Secretion of ADAMTS17 requires *O*-fucosylation and its autocatalytic activity does not depend on propeptide processing by furin. ADAMTS17 binds recombinant fibrillin-2 but not fibrillin-1 and does not cleave either. It colocalizes to fibrillin-1 containing microfibrils in cultured fibroblasts and suppresses fibrillin-2 (FBN2) incorporation in microfibrils, in part by transcriptional downregulation of *Fbn2* mRNA expression. RNA *in situ* hybridization detected *Adamts17* expression in specific structures in the eye, skeleton and other organs, where it may regulate the fibrillin isoform composition of microfibrils.

Extracellular proteases execute a plethora of functions in the formation, physiological remodeling, and pathological destruction of extracellular matrices (ECM) in most, if not all, tissues. The ADAMTS family of secreted zinc metalloproteases includes 19 enzymes, which are involved in several physiological and pathological processes^1^. Physiological processes include procollagen-I processing by ADAMTS2, which is a prerequisite for collagen fibril formation in skin, and versican clearance by ADAMTS1, 5, 9, and 20 during embryogenesis^2^,^3^. The processing of ultrahigh molecular weight forms of von-Willebrand factor by ADAMTS13 is crucial for hemostasis^4^. Among relevant pathogenic contributions, ADAMTS4 and 5 are involved in cartilage destruction in arthritis by degrading aggrecan^5^,^6^.

*ADAMTS17* encodes an “orphan” member of the ADAMTS family, i.e., one for which no substrates or biological functions are known although it has well established disease significance. Autosomal recessive *ADAMTS17* mutations cause Weill-Marchesani-like syndrome in humans (MIM #613195)^7^,^8^. Affected individuals have short stature and eye anomalies, including ectopia lentis (lens dislocation), myopia, and glaucoma. However, they lack joint stiffness, brachydactyly (short digits), or cardiac valve disease typically associated with Weill-Marchesani syndrome (WMS), which is caused by recessive *ADAMTS10* mutations (MIM #277600) or dominant *FBN1* mutations (MIM #608328)^9^,^10^,^11^. Recent identification of *ADAMTS17* mutations in a family with short-stature, ectopia lentis and brachydactyly suggests overlap of WMS and WMS-like syndrome, constituting a WMS spectrum^7^,^12^. In contrast to humans, canine *ADAMTS17* mutations cause isolated ectopia lentis with no skeletal or cardiac anomalies^13^,^14^. *ADAMTS17* was also linked to primary open angle glaucoma and to variant height in humans and dogs^15^,^16^,^17^,^18^,^19^. These genetic studies strongly linked *ADAMTS17* to *FBN1* and implicated it in the formation of the ciliary zonule in the eye, the fibrillin-based structure defective in ectopia lentis, and in the regulation of skeletal growth.

Fibrillin-1 represents one of three fibrillin isoforms in humans. Fibrillins are the core constituents of tissue microfibrils, an ECM scaffold that can include microfibril-associated glycoproteins, TGFβ superfamily proteins, latent TGFβ-binding proteins, fibulins, tropoelastin, and ADAMTS proteins, such as ADAMTS10^20^,^21^,^22^,^23^. These additional components may regulate microfibril assembly or contribute specialized functions to the microfibril scaffold. During the embryonic period, *FBN2* mRNA expression predominates, whereas *FBN1* is the dominant fibrillin mRNA isoform in adult tissues^24^,^25^. However, fibrillin-2 may persist in the core of adult microfibril bundles^26^. The transition from *Fbn2* mRNA prevalence to *Fbn1* mRNA dominance in the mouse occurs during the late embryonic to early juvenile period^25^. Because the proportion of fibrillin-1 or -2 within microfibrils may be determined by fibrillin isoform availability without a bias toward inclusion of either, the expression levels of the respective genes are likely to be important in determining microfibril composition. However, other mechanisms regulating fibrillin isoform selection may be active. For example, genetic ablation of ADAMTS-like 2 (ADAMTSL2) resulted in the accumulation of fibrillin-2 microfibrils around bronchial smooth muscle cells^27^. In addition, ADAMTS10, ADAMTSL4, and ADAMTSL6 accelerated formation of fibrillin-1 microfibrils by cultured cells, or upon overexpression in mice^28^,^29^,^30^.

Here we report the first molecular and functional characterization of ADAMTS17, which reveals important characteristics of this protease, such as autocatalytic generation of several proteoforms and identifies post-translational modifications required for ADAMTS17 secretion. In addition, we demonstrate that ADAMTS17 may have a role in regulating the fibrillin isoform composition of microfibrils in specific tissues during development.

## Results

### ADAMTS17 is autoproteolytically processed upon secretion

**Figure 1:**
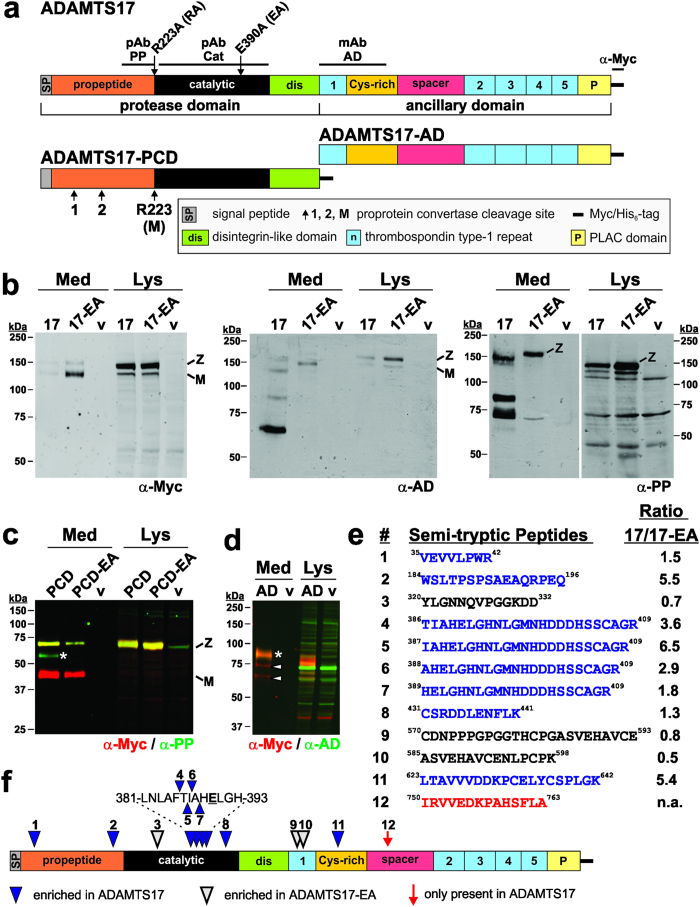
ADAMTS17 undergoes autocatalytic processing. (**a**) Domain organization of ADAMTS17 and the constructs ADAMTS17-PCD and ADAMTS17-AD. The location of the ADAMTS17 antibody epitopes (black line) and the sites of site-directed mutagenesis (furin-site: R^223^A, active site: E^390^A) are indicated above the ADAMTS17 domains. The predicted furin/PACE cleavage sites are shown below the ADAMTS17-PCD construct. Cleavage at site R223 results in mature (“M”) ADAMTS17. (**b**) Western blot analysis of conditioned medium (Med) and cell lysate (Lys) from full-length ADAMTS17 (17), the active site mutant ADAMTS17^EA^ (17-EA), and empty vector (v) expressing HEK293F cells. Western blots were probed with the indicated antibodies and detected with enhanced chemiluminescence. (**c**) Western blot analysis of conditioned medium (Med) and cell lysate (Lys) from ADAMTS17-PCD (PCD), ADAMTS17-PCD^EA^ (PCD-EA), and empty vector (v) expressing HEK293F cells. The asterisk indicates a band which is reactive with anti-propeptide antibody (anti-PP, green), but not anti-myc (red), and which is absent in ADAMTS17-PCD^EA^. (**d**) Western blot analysis of conditioned medium (Med) and cell lysate (Lys) from ADAMTS17-AD (AD) and empty vector (v) expressing HEK293F cells. The asterisk indicates intact ADAMTS17-AD (yellow), reactive with anti-myc (red) and anti-ancillary domain antibody (anti-AD, green). Arrowheads indicate species reactive with only anti-myc, but not anti-AD (red). (**e**) Semi-tryptic peptides resulting from ADAMTS17 autoproteolysis and identified by LC-MS/MS (blue: enriched in ADAMTS17, black: enriched in ADAMTS17^EA^, red: present only in wild-type ADAMTS17) (**f**) Location of semi-tryptic peptides in ADAMTS17. Numbers and color coding correspond to the list in e. mAb, monoclonal antibody; M, mature enzyme; pAb, polyclonal antibody; v, vector; Z, zymogen.

Recombinant full-length human ADAMTS17 (Fig. 1a) was detected as two molecular species in western blots of cell lysates from transiently transfected HEK293F cells, using anti-myc monoclonal antibody, as well as monoclonal and polyclonal antibodies directed to epitopes located in the ADAMTS17 ancillary domain or propeptide, respectively (Fig. 1a,b, left-hand panel). The dominant species in cell lysates corresponded to the ADAMTS17 zymogen (Z, observed MW: ~160 kDa, predicted MW: 125.3 kDa) and the minor species to mature ADAMTS17 (observed MW: ~130 kDa, predicted MW: 102.9 kDa), consistent with constitutive proprotein convertase (e.g., furin)-mediated cleavage of the propeptide (predicted MW: 22.4 kDa) (Fig. 1b, left-hand panel). The greater than predicted MW of ADAMTS17 is ascribed to glycosylation (see below). In conditioned medium of transfected cells, secreted ADAMTS17 was poorly detected with anti-myc antibody (Fig. 1b, left-hand panel). In contrast, ADAMTS17 harboring a Glu^390^Ala mutation within the active site (ADAMTS17^EA^) was readily detected in the conditioned medium by the anti-myc antibody. The Glu^390^Ala mutation targets the catalytic Glu residue and is traditionally employed to inactivate metalloproteases^31^,^32^. Hence, the apparent absence of ADAMTS17 in conditioned medium is likely due to autoproteolysis (Fig. 1b, left-hand panel). As expected, most of the secreted ADAMTS17^EA^ was processed to the mature form. ADAMTS17 autoproteolysis was also evident from detection of ADAMTS17 peptides using the monoclonal and polyclonal antibodies against the ancillary domain and the propeptide, respectively, which were not detected in the conditioned medium of the ADAMTS17^EA^ mutant (Fig. 1b, center and right-hand panels). Absence of specific ADAMTS17 cleavage products in cell lysates (other than the furin-processed form), suggested that ADAMTS17 underwent autoproteolysis after secretion. Since the C-terminal anti-myc reactivity was lost due to autoproteolysis of secreted wild-type ADAMTS17, and a cleavage product smaller than the zymogen but larger then mature ADAMTS17 (~150 kDa) was observed with the antibody against the propeptide, one of the autoproteolytic cleavage sites must be located close to the ADAMTS17 C-terminus. However, this putative C-terminal fragment was neither detected in western blots nor by mass spectrometry (see below). The specificity of ADAMTS17 antibodies was demonstrated by the lack of reactive bands in medium and cell lysate from cells transfected with the empty vector (Fig. 1b, lanes marked by v). A similar contrasting fragmentation of ADAMTS17 and ADAMTS17^EA^ was observed after transfection of ADAMTS17 in COS-1 cells (data not shown), supporting the observed autoproteolysis of ADAMTS17.

In contrast to full-length ADAMTS17, a C-terminally truncated construct, ADAMTS17-PCD (Fig. 1a), was readily detectable in the medium of transfected HEK293F cells using anti-myc antibody (Fig. 1c, red/yellow). The observed MW of the major species corresponded to the zymogen (Z, observed MW: ~65 kDa, predicted MW: 61.3 kDa, yellow) and the mature form (M, observed MW: ~40 kDa, predicted MW: 38.9 kDa, red only). The ADAMTS17-PCD zymogen, but not its mature form, was detected with the polyclonal propeptide antibody (green/yellow). Appearance of an anti-propeptide antibody reactive band (~55 kDa) in ADAMTS17-PCD which was unreactive with anti-myc and absent in the ADAMTS17-PCD^EA^ construct suggested limited autoproteolysis of ADAMTS17-PCD (Fig. 1c, asterisk, green only). The fluorescent western blotting technique used here and in the following experiments allows for the simultaneous detection of two antibody epitopes on the same membrane. Therefore, protein species specifically reacting with either one of the antibodies appear red or green. Protein species reacting with both antibodies appear yellow.

A construct expressing the ADAMTS17 ancillary domain (ADAMTS17-AD, Fig. 1a) was also readily detected on western blots of medium with anti-myc and with the ancillary domain antibody (observed MW: ~85 kDa, predicted MW: 67.2 kDa) (Fig. 1d, yellow). Additional bands in ADAMTS17-AD containing medium observed with anti-myc (~73 kDa, 65 kDa, Fig. 1d, red), but unreactive with the ADAMTS17 monoclonal antibody indicated fragmentation near the N-terminus of ADAMTS17-AD by an ambient protease, with loss of the epitope for the monoclonal antibody. In both empty vector and ADAMTS17-AD transfected cell lysates but not the medium, multiple bands reactive with the monoclonal antibody were observed, indicating cross reactivity with unknown proteins (Fig. 1d, green).

To identify ADAMTS17 autocatalytic processing sites, we compared liquid chromatography tandem mass spectra (LC-MS/MS) of conditioned medium from cells expressing ADAMTS17 or ADAMTS17^EA^. We detected twelve semi-tryptic peptides (i.e. peptide sequences with one tryptic cleavage site and one non-tryptic cleavage site arising from ADAMTS17 autoproteolysis) (Fig. 1e,f, Supplemental Table 1 and Supplemental Fig. 1). One peptide was uniquely present in ADAMTS17 medium, and eight were enriched in ADAMTS17 medium, suggesting autoproteolysis, whereas only three peptides were enriched in ADAMTS17^EA^ medium. Taken together, western blotting results comparing ADAMTS17 with ADAMTS17^EA^ and LC-MS/MS data unequivocally indicate that wild type ADAMTS17 is secreted and autocatalytically processed at multiple sites (Fig. 1f). Secondary processing by ambient proteases, or proteases activated by ADAMTS17 may also contribute to the fragmentation pattern, but remain to be characterized.

### ADAMTS17 autoproteolysis occurs *in trans* and does not require the ancillary domain or furin processing

**Figure 2:**
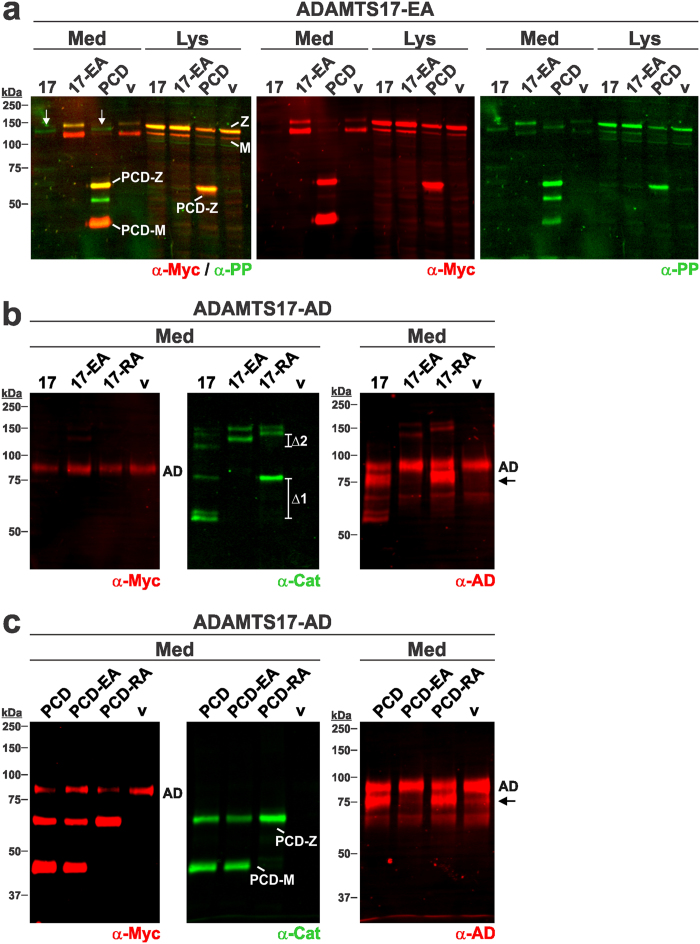
ADAMTS17 undergoes autocatalysis *in trans* that is independent of furin processing. (**a**) Western blot analysis of conditioned medium (Med) and cell lysate (Lys) of HEK293F cells co-transfected with ADAMTS17^EA^ (17-EA) and ADAMTS17 (17), ADAMTS17^EA^ (17-EA), ADAMTS17-PCD (PCD), or empty vector (v) indicate trans-cleavage of ADAMTS17^EA^ by ADAMTS17 and ADAMTS17-PCD. White arrows indicate lanes with cleavage products, detected with the anti-propeptide antibody (anti-PP, green). Anti-myc (red) detects zymogen and mature forms of ADAMTS17 in the cell lysates and zymogen and mature form of ADAMTS17-PCD in the cell lysate and conditioned medium. (**b**) Western blot analysis of conditioned medium (Med) from HEK293F cells, stably expressing ADAMTS17-AD (AD) and transiently expressing ADAMTS17 (17), ADAMTS17^EA^ (17-EA), ADAMTS17^RA^ (17-RA) or empty vector (v). Note reduced levels of ADAMTS17-AD in the presence of ADAMTS17 or ADAMTS17^RA^ (left-hand panel, red) and mass shift of furin-resistant species compared to wild type (brackets indicated by Δ1 and Δ2) (middle panel, green). The proteolytic product detected with anti-AD is indicated with an arrow (right-hand panel, red) and only appears upon transfection of ADAMTS17-AD expressing cells with ADAMTS17 or ADAMTS17^RA^. (**c**) Western blot analysis of conditioned medium (Med) from HEK293F cells, stably expressing ADAMTS17-AD (AD) and transiently expressing ADAMTS17-PCD (PCD), ADAMTS17-PCD^EA^ (PCD-EA), ADAMTS17-PCD^RA^ (PCD-RA) or empty vector (v). The mature form of ADAMTS17-PCD (PCD-M) was not generated in ADAMTS17-PCD^RA^, indicating lack of furin processing (left-hand panel, red and middle panel, green). Furin processing did not abolish proteolytic activity as observed by reduction in the intensity of anti-myc reactive ADAMTS17-AD (left-hand panel, top band) or the appearance of an additional band reactive with the anti-AD antibody (arrow, right panel, red). Note that the α-Myc antibody (red) detects both ADAMTS17-PCD and ADAMTS17-AD. M, mature enzyme; Z, zymogen.

To determine whether ADAMTS17 autoproteolysis occurred *in cis* or *in trans*, we co-transfected HEK293F cells with plasmids expressing ADAMTS17^EA^ plus ADAMTS17, ADAMTS17^EA^, ADAMTS17-PCD, or empty vector control, respectively (Fig. 2a). ADAMTS17^EA^ was detected at reduced levels in conditioned medium upon co-expression with ADAMTS17 or ADAMTS17-PCD. Since the Glu^390^Ala mutation abolishes the proteolytic activity of ADAMTS17, the findings indicate that proteolysis of ADAMTS17^EA^ was mediated by co-transfected ADAMTS17 or ADAMTS17-PCD, which could be described as autoproteolysis *in trans* (Fig. 2a). Proteolysis of ADAMTS17^EA^ by ADAMTS17-PCD suggested that the ancillary domain was not required for ADAMTS17^EA^ proteolysis.

To determine whether furin processing of ADAMTS17 was required for autoproteolysis, we introduced an Arg223Ala mutation at the P1 residue within the furin recognition sequence ^220^RERR^223^ in ADAMTS17 and ADAMTS17-PCD (Fig. 1a). The resulting retarded in-gel migration (Fig. 2b, middle panel, green, Δ1 and Δ2, respectively) reflects retention of the propeptide in the furin-resistant ADAMTS17^RA^ mutant constructs. Furin-resistance of ADAMTS17-PCD^RA^ was demonstrated by the absence of mature ADAMTS17-PCD using the anti-catalytic domain antibody (Fig. 2c, middle panel, green).

We transfected the furin-resistant constructs into the ADAMTS17-AD stable cell line. Anti-myc reactivity of ADAMTS17-AD was reduced in the presence of ADAMTS17 and ADAMTS17^RA^, but not ADAMTS17^EA^ (Fig. 2b, left-hand panel, red). Using the monoclonal ADAMTS17 antibody, we observed a band at ~75 kDa, which was only present upon proteolytic processing of ADAMTS17-AD by ADAMTS17 and ADAMTS17^RA^ (Fig. 2b, right-hand panel, red, arrow), suggesting proteolytic activity despite the presence of the propeptide. Similar results were obtained upon transfection of ADAMTS17-PCD, ADAMTS17-PCD^EA^, or ADAMTS17-PCD^RA^ into stable ADAMTS17-AD expressing cells (Fig. 2c). ADAMTS17-PCD and ADAMTS17-PCD^RA^, but not the active site mutant ADAMTS17-PCD^EA^, cleaved ADAMTS17-AD, demonstrated by reduced reactivity with anti-myc (Fig. 2c, left-hand panel, red) and appearance of the ~75 kDa band detected with the monoclonal ADAMTS17 antibody (Fig. 2c, right-hand panel, arrow, red). Thus, furin processing of the ADAMTS17 propeptide is not required for ADAMTS17 activation. We conclude that the domains contained in ADAMTS17-PCD are necessary and sufficient for the intermolecular interaction required for full-length ADAMTS17 autoproteolysis *in trans* and that the cleaved target sequences are located within the ancillary domain and the protease domain of full-length ADAMTS17.

### ADAMTS17 is N-glycosylated and its secretion requires *O*-fucosylation of thrombospondin type-1 repeats

**Figure 3:**
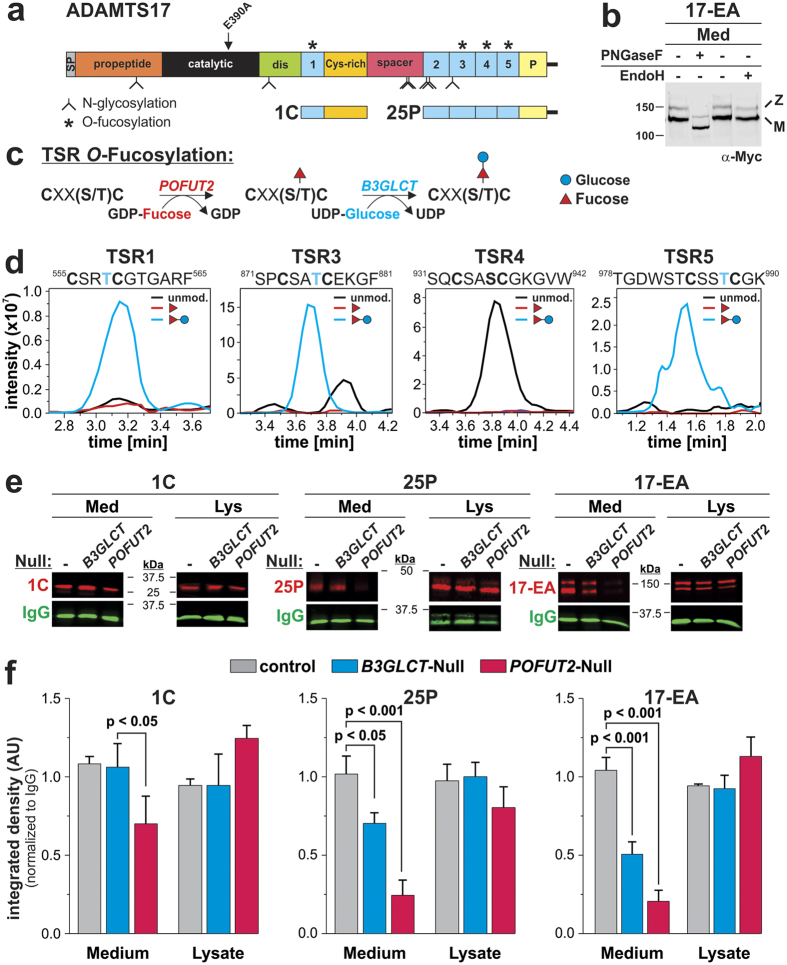
ADAMTS17 is *N*-glycosylated and *O*-fucosylated and requires *O*-fucosylation for secretion. (**a**) Predicted N-glycosylation (branched stems) and *O*-fucosylation sites (asterisks) in ADAMTS17 and constructs used for LC-MS/MS and the ADAMTS17 secretion analysis. (**b**) Western blot analysis of ADAMTS17^EA^-containing medium (Med) with or without treatment with peptide-N-glycosidase F (PNGaseF) or endoglycosidase H (EndoH). M = mature enzyme; Z = zymogen. For the full-length western blot see Supplemental Fig. 5a. (**c**) Schematic of TSR *O*-fucosylation depicting the enzymes and substrates involved. (**d**) Extracted ion chromatograms of the ions corresponding to unmodified (black), *O*-fucose (red), and *O*-fucose-glucose (blue) glycoforms of peptides from ADAMTS17 TSRs as identified by nano-LC-MS/MS. The *O*-fucosylation consensus sequence of the respective TSR and the modified residue (blue) are indicated above each chromatogram. (**e**) Western blot analysis of medium (Med) and lysate (Lys) from HEK293T cells with inactivated *B3GLCT* or *POFUT2* (null) and transiently transfected with ADAMTS17-1C (1C), ADAMTS17-25P (25P) and ADAMTS17^EA^ (red). Co-transfected IgG (green) was used as the secretion control for quantification since it does not contain TSRs. For the full-length western blots see Supplemental Fig. 5b,c. (**f**) Quantification of the integrated densities of the respective bands normalized to IgG (n = 3). Statistical significance was calculated using a two-sided Student t-test and compared to the band intensity measured after secretion from wild-type cells.

ADAMTS17 contains seven predicted N-glycosylation sites, of which five are located in the ancillary domain (Fig. 3a). Digestion of ADAMTS17^EA^ with PNGase F resulted in more rapid migration of the zymogen and mature forms under reducing conditions, indicating N-glycosylation (Fig. 3b). In contrast, secreted ADAMTS17^EA^ was insensitive to endoglycosidase H, suggesting the N-glycans are of the complex or hybrid type (Fig. 3b).

The thrombospondin type 1 repeats (TSRs) of ADAMTS proteins are potential targets for post-translational modification by *O*-fucosylation, which adds a Glucose-β1–3Fucose disaccharide to the consensus sequence Cys-Xaa-Xaa-Ser/Thr-Cys (Xaa is any amino acid, the modified residue is underlined) (Fig. 3c)^33^. Four of five TSRs in ADAMTS17 carry the required consensus sequence (Fig. 3a). Recombinant ADAMTS17 constructs 1C and 25P (Fig. 3a) were purified to homogeneity from transiently transfected HEK293F cells for analysis by LC-MS/MS. We detected the Glucose-β1–3Fucose disaccharide on TSR1, 3, and 5, but not TSR4 (Fig. 3d, Supplemental Fig. 2, Supplemental Table 2). *O*-Fucosylation is implicated in quality control of proteins containing TSRs^34^,^35^. To determine whether lack of or incomplete *O*-fucosylation affected the secretion of ADAMTS17, we transfected the ADAMTS17 constructs 1C, 25P, and ADAMTS17^EA^ into HEK293F cells with inactivated POFUT2 or B3GLCT, the enzymes attaching the fucose and glucose moiety, respectively, to the serine/threonine residue. In the absence of *POFUT2*, secretion of 1C into the medium was significantly reduced and secretion of 25P and ADAMTS17^EA^ was nearly abolished (Fig. 3e,f, red channel). *B3GLCT* knockout had no effect on the secretion of 1C, but significantly reduced secretion of 25P and ADAMTS17^EA^ correlating with the presence of 1, 2, or 3 *O*-fucosylation sites, respectively. The levels of the ADAMTS17 constructs in the cell lysates were not affected by the absence of *POFUT2* or *B3GLCT*. Together, these results show extensive glycosylation of ADAMTS17 and its functional impact on ADAMTS17 secretion.

### ADAMTS17 forms multimers via intermolecular disulfide bonds

**Figure 4:**
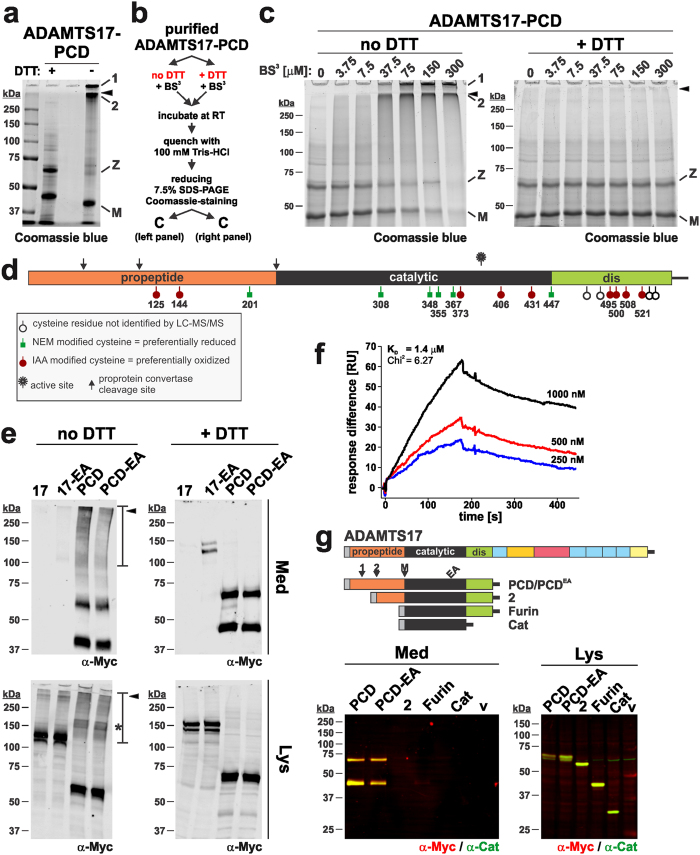
ADAMTS17-PCD self-associates via disulfide bonds involving the propeptide. (**a**) Coomassie blue stained 7.5% SDS-PAGE of purified ADAMTS17-PCD (20 μg/lane) separated under reducing (+ DTT) and non-reducing (− DTT) conditions. The black arrowhead depicts the border between the stacking and separating gel; 1 and 2 indicate large molecular weight complexes of ADAMTS17-PCD, which in the absence of DTT, did not enter the stacking or separating gel, respectively. The “+ DTT” and “− DTT” lanes are separated by an empty lane. M, mature enzyme; Z, zymogen. (**b**) Flow chart outlining the design of cross-linking experiments in c. (**c**) Coomassie blue-stained reducing SDS-PAGE of purified ADAMTS17-PCD (10 μg/lane) incubated with increasing concentrations of BS^3^ cross-linker in the presence (+ DTT) or absence of reducing agent (no DTT) (Annotations as in a). (**d**) Schematic of ADAMTS17-PCD showing cysteine residues and indicating their oxidized or reduced status determined by LC-MS/MS. (**e**) Western-blot analysis of conditioned medium (Med) and cell lysate (Lys) from cells expressing ADAMTS17 (17), ADAMTS17^EA^ (17-EA), ADAMTS17-PCD (PCD), and ADAMTS17-PCD^EA^ (PCD-EA) under reducing (+ DTT) and non-reducing conditions (no DTT). Areas of the gel in the high MW range (>100 kDa) reacting with anti-myc in the absence of DTT in medium and lysate are outlined with a bar. The asterisk indicates prominent anti-myc reactive ADAMTS17-PCD band in the lysate under non-reducing conditions. (**f**) Surface plasmon resonance indicates interaction of ADAMTS17-PCD to surface immobilized ADAMTS17-PCD in a dose dependent manner. The dissociation constant (K_D_) was calculated assuming 1:1 binding. (**g**) Schematic of ADAMTS17-PCD truncation mutants (top panel). Western blot analysis of conditioned medium and cell lysate from HEK293 cells transiently transfected with ADAMTS17-PCD truncation mutants indicates anti-myc and anti-catalytic domain antibody reactive bands (yellow) of the predicted molecular weight in cell lysates for each mutant (bottom, right-hand panel). However, the entire propeptide is required for secretion (bottom, left-hand panel).

Electrophoresis of purified ADAMTS17-PCD under reducing and non-reducing conditions followed by Coomassie blue staining demonstrated that mature ADAMTS17-PCD migrated more rapidly under non-reducing conditions (Fig. 4a), consistent with a globular conformation in the presence of disulfide bonds. Under non-reducing conditions, however, the ADAMTS17-PCD zymogen band was considerably weaker than the mature form and high MW bands were evident, indicative of disulfide-bonded multimers (Fig. 4a). The disproportionate shift of the zymogen, but not the processed catalytic domain under non-reducing conditions suggested that the propeptide may mediate the formation of ADAMTS17-PCD multimers.

The formation of multimers by ADAMTS7-PCD was further investigated by incubating purified ADAMTS17-PCD with bis(sulfosuccinimidyl)suberate (BS^3^), a non-reducible bifunctional amine-reactive cross-linker with a 11.4 Å spacer in the presence or absence of DTT (Fig. 4b), followed by electrophoresis under reducing conditions and Coomassie blue staining (Fig. 4c). In the absence of DTT, we observed a BS^3^ concentration-dependent electrophoretic shift of the zymogen and mature forms of ADAMTS17-PCD to the upper regions of the gel (Fig. 4c, left-hand panel). In the presence of DTT, which reduces disulfide bonds, these high MW bands were absent. Incubation of bovine serum albumin (BSA) with BS^3^ under the same experimental conditions did not alter its electrophoretic mobility (data not shown). This experiment, together with the differential migration of the zymogen and the mature ADAMTS17-PCD under reducing versus non-reducing conditions suggests that the oligomers arise by disulfide bond formation, but other residues of ADAMTS17-PCD are in sufficient proximity for experimental BS^3^-mediated cross-linking.

The catalytic and disintegrin-like domains of ADAMTS proteases contain eight cysteine residues each, shown to form four disulfide bonds within each domain by high-resolution structures of ADAMTS1, 4, and 5^36^,^37^,^38^. In contrast, neither structural information nor disulfide bond mapping is available for ADAMTS propeptides. Most ADAMTS propeptides, including ADAMTS17, contain three cysteine residues, suggesting that one unpaired cysteine is available for intermolecular disulfide-bond formation. We probed for free cysteines in purified ADAMTS17-PCD, using an LC-MS/MS approach. Free and solvent-accessible cysteines in ADAMTS17-PCD were labeled with N-ethylmaleimide (NEM) prior to reduction, alkylation with iodoacetamide (IAA), and proteolytic digestion. LC-MS/MS identified peptides containing six cysteine residues that were NEM-modified, indicating at least partial availability for disulfide-bonding (Fig. 4d, Supplemental Fig. 3, Supplemental Table 3). Five of the NEM-modified cysteine residues were located in the catalytic domain and Cys^201^ was located in the propeptide. The two other cysteine residues present in the propeptide (Cys^125^ and Cys^144^) were detected only in the IAA-modified form, indicating that they were not accessible to NEM or were oxidized, i.e., engaged in disulfide bonds. To exclude the possibility that ADAMTS17-PCD oligomerization was an artifact arising from purification and concentration of the protein, we analyzed whether oligomerization was present in conditioned medium of cells expressing ADAMTS17^EA^ or ADAMTS17-PCD (Fig. 4e). Our data suggest that in the absence of DTT, high MW ADAMTS17^EA^ species reacting with anti-myc are present in the medium.

We used ADAMTS17-PCD as both the analyte and immobilized ligand in surface plasmon resonance (SPR) and found that it could bind to itself with a characteristic SPR binding curve and a dissociation constant (K_D_) of 1.4 μM (Fig. 4f). Taken together, the findings suggested that secreted PCD formed high MW multimers through a combination of non-covalent interactions, as detected by SPR, and disulfide bond formation, most likely involving Cys^201^ within the propeptide.

To elucidate the role of the ADAMTS17 propeptide in ADAMTS17 secretion, we sequentially truncated the propeptide at the second and third furin processing site respectively (Fig. 4g). When these N-terminally truncated ADAMTS17 constructs encoding either the catalytic domain alone or the catalytic domain and the disintegrin-like domain were transfected into HEK cells, they were detected in cell lysate as bands of the predicted molecular mass, but were not detected in the conditioned medium (Fig. 4g, yellow due to simultaneous reactivity with anti-myc, red, and anti-Cat, green). This suggests that inclusion of the propeptide is crucial for effective secretion of ADAMTS17.

### Tissue-specific *Adamts17* mRNA expression during mouse embryonic development

**Figure 5:**
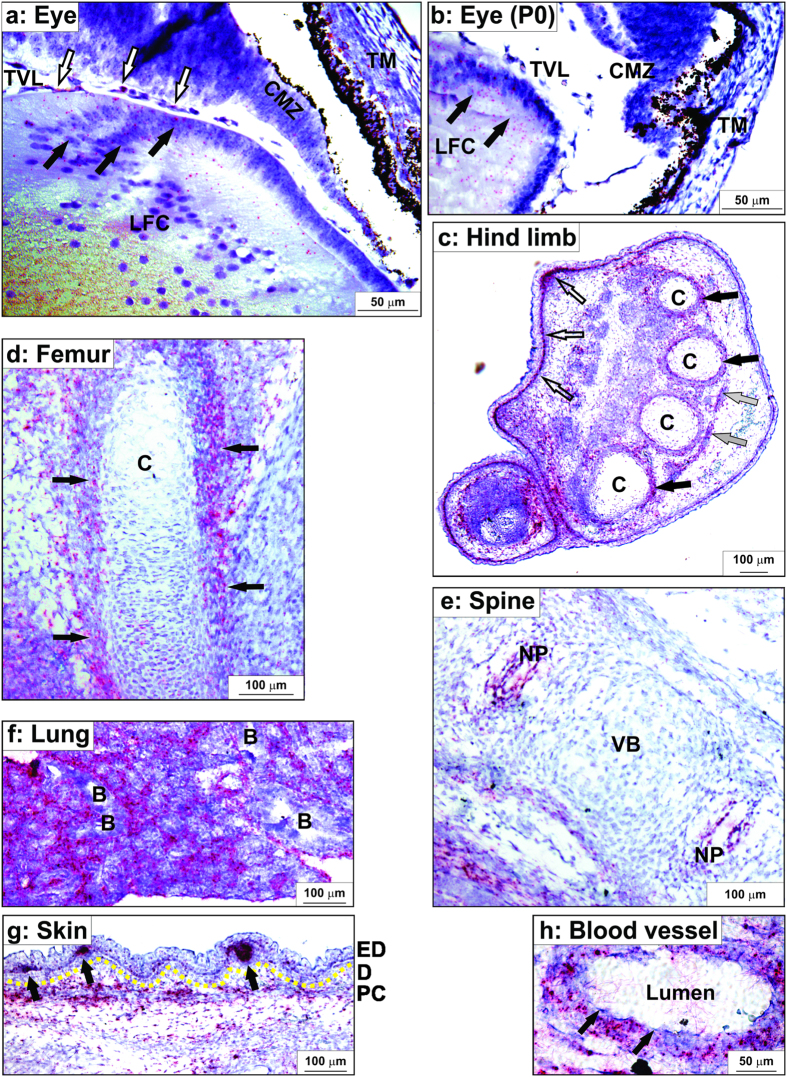
*Adamts17 in situ* hybridization in 16.5 day-old mouse embryos or neonates showing expression in tissues relevant to Weill-Marchesani syndrome. The RNAScope method results in red signal over cells containing *Adamts17* mRNA. Sections were counterstained with hematoxylin (blue). (**a**,**b**) At embryonic day (E) 16.5 (**a**), *Adamts17* was expressed in equatorial lens fiber cells (LFC, black arrows), capillaries of the tunica vasculosa lentis (TVL, white arrows), the non-pigmented epithelium of the ciliary margin zone (CMZ), and the trabecular meshwork cells (TM) of the eye. In neonates (**b**) *Adamts17* continued to be expressed in LFC, but expression was reduced in the other ocular tissues. (**c**–**e**) *Adamts17* mRNA expression in the skeleton. In the hind limb autopod (**c**), *Adamts17* was detected in the perichondrium around cartilage (C, black arrows), in tendon (grey arrows), and in the dermis and hair follicles (white arrows). *Adamts17* was expressed in the perichondrium of the femur (**d**) (arrows) and in the nucleus pulposus (NP) of the intervertebral disks but not in the vertebral bodies (VB) (**e**). (**f**) Strong *Adamts17* mRNA expression was found in the lung parenchyma, but not the bronchial epithelium (B). (**g**) *Adamts17* mRNA expression in skin was localized to the hair follicles and the panniculus carnosus (PC). A yellow line marks the dermal (D) – epidermal (ED) junction. Arrows indicate developing hair follicles. (**h**) In blood vessels, *Adamts17* was expressed in the smooth muscle cells of the blood vessel wall, but not endothelial cells (arrows).

The distribution of *Adamts17* mRNA was analyzed by *in*-*situ* hybridization in sections from embryonic day (E) 16.5 old mouse embryos and from neonates (Fig. 5). In the eye at E16.5 (Fig. 5a), the strongest *Adamts17* expression was detected in lens fiber cells at the lens equator, the site of the insertion of the ciliary zonule which is defective in ectopia lentis, in the non-pigmented epithelium of the ciliary body, which is the other attachment site of the zonule, and in the trabecular meshwork, which if blocked, can lead to glaucoma. We also detected expression in the blood vessels of the tunica vasculosa lentis and in the corneal stroma. In neonatal eyes (Fig. 5b), *Adamts17* expression continued strongly in lens fiber cells, but was reduced in other ocular components expressing *Adamts17* at E16.5. In long bones, *Adamts17* expression was seen in the perichondrium surrounding the cartilage of long bone primordia, such as the metacarpals, metatarsals (Fig. 5c), and the femur (Fig. 5d), but not in the growth plate cartilage (Fig. 5d). In addition, we detected *Adamts17* expression in developing tendons (Fig. 5c). In the intervertebral disc we observed *Adamts17* expression in the nucleus pulposus (Fig. 5e). The strongest *Adamts17* mRNA expression was observed diffusely in lung parenchyma, but was absent from the bronchial epithelium (Fig. 5f), and was shown to be specific using a control RNA probe (data not shown). In the skin, *Adamts17* was expressed in the epidermal basal cell layer, the underlying dermal mesenchyme and intensely in developing hair follicles (Fig. 5g). In blood vessels, *Adamts17* mRNA was restricted to smooth muscle cells of the tunica media, but was absent from the endothelium (Fig. 5h). Notably, fibrillins are expressed at each of these sites^25^,^27^,^39^.

### ADAMTS17 interacts with fibrillin-2 but not fibrillin-1 or fibronectin, and does not cleave fibrillin-1 or fibrillin-2

**Figure 6:**
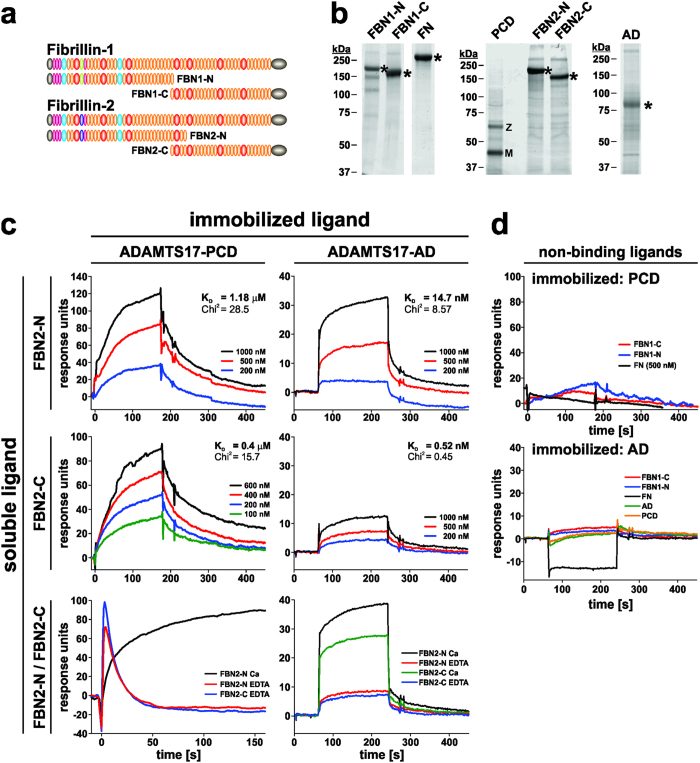
ADAMTS17-PCD binds to fibrillin-2 but not fibrillin-1 or fibronectin. (**a**) Domain structure of recombinant fibrillin-1 and fibrillin-2 peptides used to analyze interactions with ADAMTS17-PCD (PCD) or ADAMTS17-AD (AD). (**b**) Coomassie blue stained SDS-PAGE showing the integrity and purity of the recombinant proteins used in the interaction studies. The asterisks indicate the full-length constructs. M, mature enzyme; Z, zymogen. (**c**) Surface plasmon resonance shows binding of FBN2-N and FBN2-C to ADAMTS17-PCD (left-hand panels) or ADAMTS17-AD (right-hand panels). Binding was dose-dependent and Ca^2+^-dependent. The dissociation constant (K_D_) was calculated assuming 1:1 stoichiometry. (**d**) ADAMTS17-PCD (top panel) or ADAMTS17-AD (bottom panel) did not bind to FBN1-N, FBN1-C, or cellular fibronectin (FN) analytes and ADAMTS17-AD did not self-interact or bind to ADAMTS17-PCD.

Because of the genetic association of *ADAMTS17* with *FBN1* revealed by WMS spectrum-causing mutations, we asked whether ADAMTS17 bound to or cleaved recombinant fibrillin-1 and -2 (Fig. 6a,b)^7^,^11^,^12^,^40^. Unexpectedly, analysis of ADAMTS17-PCD binding to recombinant fibrillin fragments using SPR showed that ADAMTS17-PCD bound to the N- and C-terminal halves of FBN2 (Fig. 6c, left-hand panels), but not to FBN1 or full-length cellular fibronectin (Fig. 6d, top panel). The calculated affinities for the ADAMTS17-PCD/FBN2 interactions were in the low μM range, assuming a 1:1 stoichiometry, and the interaction was Ca^2+^-dependent (Fig. 6c, bottom left panel). We observed a similar selectivity and Ca^2+^- dependency for ADAMTS17-AD binding to FBN2 (Fig. 6c, right-hand panels). However, the calculated affinities for the ADAMTS17-AD/FBN2 interactions were in the low nM range indicating much stronger binding of ADAMTS17-AD to FBN2 compared to the ADAMTS17-PCD. ADAMTS17-AD did not bind to FBN1 or fibronectin, and did not self-interact nor bind to the ADAMTS17-PCD peptide (Fig. 6d, bottom panel). These findings suggest at least two FBN2 binding sites within ADAMTS17, with the ancillary domain conferring the strongest binding to FBN2.

**Figure 7:**
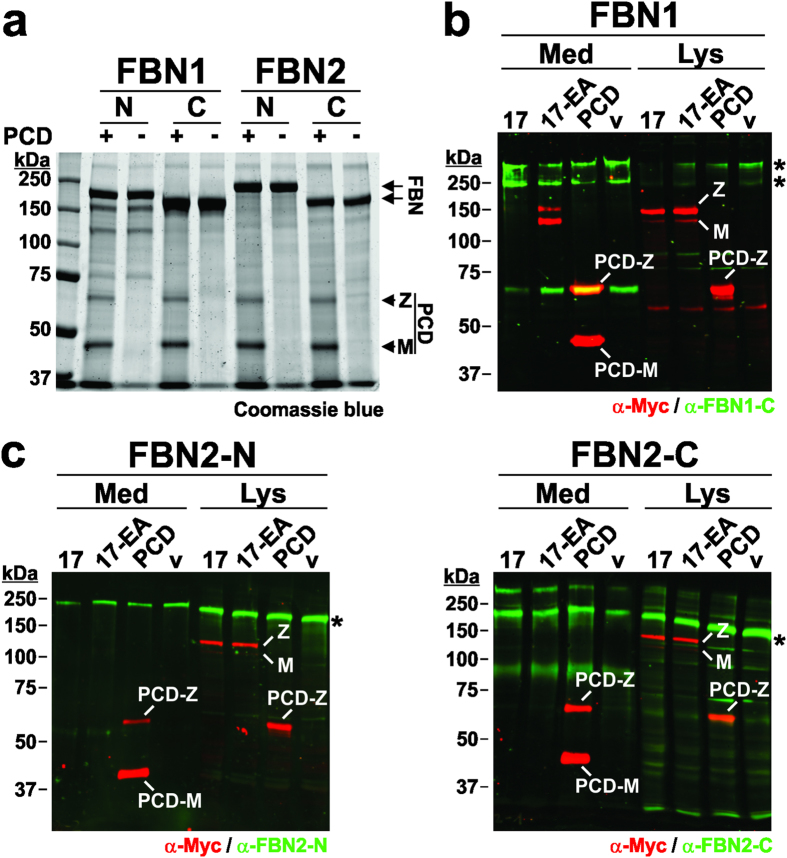
ADAMTS17-PCD does not cleave fibrillin-1 or fibrillin-2. (**a**) Coomassie-blue stained SDS-PAGE of fibrillin-1 (FBN1-N, FBN1-C) and fibrillin-2 (FBN2-N, FBN2-C) peptides (5 μg/lane) (see Fig. 6a) incubated with (+) or without (−) recombinant ADAMTS17-PCD (5 μg/lane) shows no evidence for proteolysis of fibrillin-1 or fibrillin-2 *in vitro*. M = mature enzyme; Z = zymogen. (**b**) Western blot analysis of conditioned medium (Med) and cell lysate (Lys) from HEK293F cells stably expressing fibrillin-1 and transiently expressing ADAMTS17 (17), ADAMTS17^EA^ (17-EA), ADAMTS17-PCD (PCD) or empty vector (v). Western blots were probed with antibodies against fibrillin-1 (anti-FBN1-C, green) and the recombinant ADAMTS17 peptides (anti-myc, red). (**c**) Western blot analysis of conditioned medium (Med) and cell lysate (Lys) from HEK293F cells stably expressing FBN2-N or FBN2-C with ADAMTS17 (17), ADAMTS17^EA^ (17-EA), ADAMTS17-PCD (PCD), or empty vector (v) shows no evidence of cleavage. Western blots were probed with antibodies detecting fibrillin-2 (anti-FBN2-N or anti-FBN2-C, green) and the recombinant ADAMTS17 peptides (anti-myc, red).

To ask whether ADAMTS17 cleaved fibrillin-1 or fibrillin-2, we incubated recombinant fibrillin polypeptides (Fig. 6a,b) with purified ADAMTS17-PCD and visualized the proteins by Coomassie staining after SDS-PAGE (Fig. 7a). No fragmentation of any of the fibrillin peptides was evident. In this assay, we used purified ADAMTS17-PCD, which showed catalytic activity (Fig. 2), because full-length ADAMTS17 could not be purified owing to its autoproteolysis. However, because the greater binding affinity of the ancillary domain suggested that ADAMTS17 ancillary domain was required for the recognition and cleavage of the fibrillin substrates, we transfected cell lines stably expressing full-length fibrillin-1 (FBN1), FBN2-N, or FBN2-C with ADAMTS17, ADAMTS17^EA^ or ADAMTS17-PCD, respectively. Conditioned medium and cell lysate were analyzed by western blotting using antibodies detecting the fibrillins (green) and recombinant ADAMTS17 proteins (red) (Fig. 7b,c). We found no evidence for cleavage of fibrillin-1 or -2 by ADAMTS17, either as reduction of the signal intensity for the intact fibrillin bands (Fig. 7b,c, asterisks) or by appearance of fibrillin fragments. These results indicate that ADAMTS17 binds selectively to fibrillin-2, but does not cleave either fibrillin isoform.

### Recombinant ADAMTS17 binds to microfibrils formed by cultured fibroblasts, with differential effects on fibrillin-1 and fibrillin-2 assembly into microfibrils

**Figure 8:**
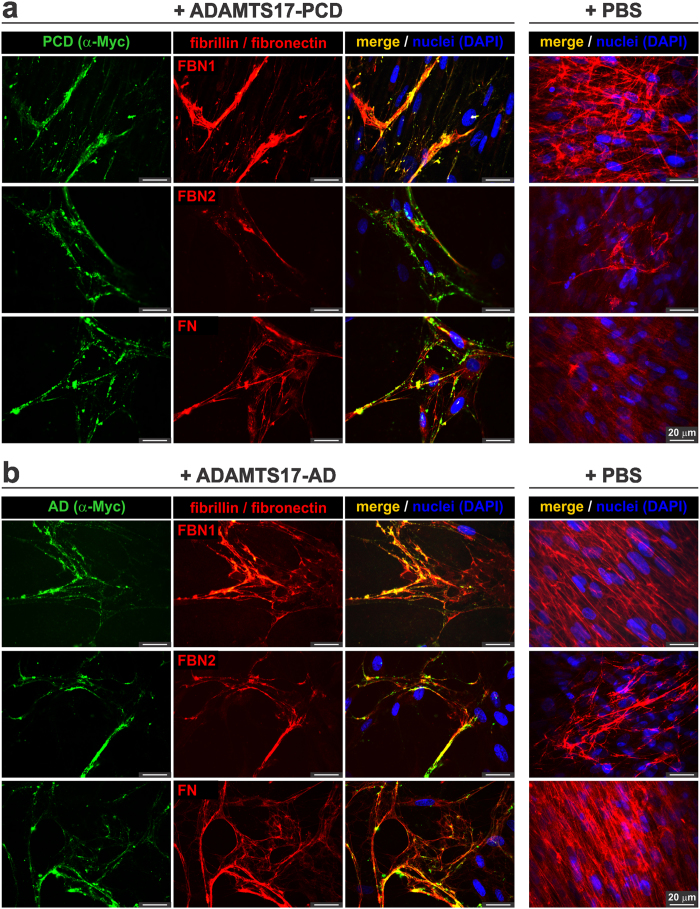
ADAMTS17-PCD and ADAMTS17-AD localize to fibrillin microfibrils deposited by cultured human dermal fibroblasts (HDF). (**a**,**b**) HDF were cultured in the presence of 50 μg ADAMTS17-PCD (**a**) or 50 μg ADAMTS17-AD (**b**) for 6 days and co-stained with anti-myc (for ADAMTS17-PCD (PCD) or ADAMTS17-AD (AD)) and antibodies against fibrillin-1 (FBN1), fibrillin-2 (FBN2), or fibronectin (FN) as indicated. Since background staining with the anti-myc antibody was very low, only the merged images are shown for the control cells (right-hand panels).

We investigated the ECM localization of ADAMTS17 in relation to fibrillin microfibrils assembled by cultured human dermal fibroblasts (HDFs)^28^,^29^. The cell number was reduced upon addition of ADAMTS17-PCD and ADAMTS17-AD to HDFs. Despite this, microfibrils were formed in these cultures, and we found that ADAMTS17-PCD (Fig. 8a) and ADAMTS17-AD (Fig. 8b) co-localized with FBN1 and FBN2 positive microfibrils. This was a surprising finding given that both ADAMTS17 peptides selectively bound to recombinant fibrillin-2, but not fibrillin-1 (Fig. 6c,d), but could be explained by the binding to heteropolymeric microfibrils containing both fibrillin-1 and fibrillin-2^41^,^42^. Since both fibrillins co-localize with fibronectin and require fibronectin for microfibril assembly in the ECM^41^,^43^, co-localization of the ADAMTS17 peptides with fibronectin was also seen (Fig. 8a,b).

**Figure 9:**
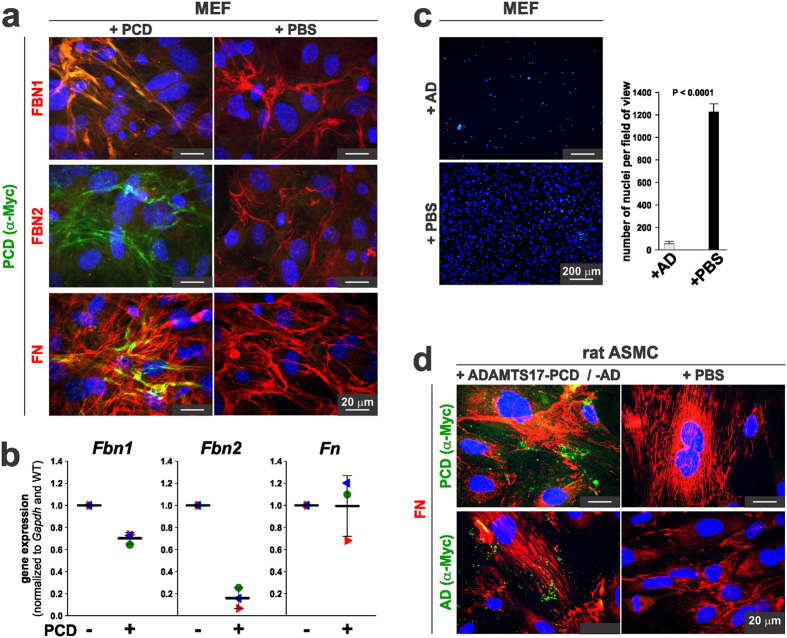
ADAMTS17-PCD interferes with fibrillin-2 microfibril formation by mouse embryo fibroblasts. (**a**) Mouse embryo fibroblasts (MEFs) were cultured in the presence of 50 μg ADAMTS17-PCD (PCD) or an equivalent volume of buffer (PBS) for 48 h and co-stained for ADAMTS17-PCD with anti-myc and with antibodies against fibrillin-1 (FBN1), fibrillin-2 (FBN2), or fibronectin (FN) as indicated. (**b**) qRT-PCR analysis of *Fbn1, Fbn2*, and *Fn* in MEFs shows reduction in *Fbn2* gene expression in the presence of ADAMTS17-PCD (PCD). Note the lesser reduction in *Fbn1* expression and no change in *Fn* gene expression. (**c**) MEF cell number was greatly reduced in the presence of ADAMTS17-AD. (**d**) Rat aortic smooth muscle cells (ASMC, A7r5) were cultured in the presence of ADAMTS17-PCD (PCD), ADAMTS17-AD (AD) or the equivalent volume of PBS for 72 h and costained using anti-myc and fibronectin (FN). Note the punctate staining of ADAMTS17-PCD and ADAMTS17-AD, which does not co-localize with fibronectin fibrils.

We used mouse embryo fibroblasts (MEFs) as a second cell type from a different species to confirm the ADAMTS17 localization results obtained in HDFs, as well as to investigate potential colocalization with fibrillin-2 microfibrils, which are made reliably by MEFs^41^. After culturing MEFs in the presence of purified ADAMTS17-PCD, immunostaining detected recombinant ADAMTS17-PCD associated with fibrillin-1 (Fig. 9a, top row). Surprisingly, few fibrillin-2 microfibrils were seen in ADAMTS17-PCD treated MEF cultures (Fig. 9a, middle row), but ADAMTS17-PCD was still localized as fibrils in the ECM. The reduction in fibrillin-2 could be explained by the stronger reduction of *Fbn2* mRNA upon addition of ADAMTS17-PCD compared to *Fbn1* mRNA (Fig. 9b). As with HDFs, recombinant ADAMTS17-PCD co-localized with fibronectin in MEF cultures, consistent with a demonstrated overlap between fibrillin-1 and fibronectin staining in cultured fibroblasts^43^. Considerable reduction of the number of adherent MEFs was observed after addition of ADAMTS17-AD (Fig. 9c), precluding microfibril staining.

To determine whether the apparent co-localization of ADAMTS17 with fibronectin represented localization to overlying microfibrils, we performed similar co-staining in rat aortic smooth muscle cells, which do not form detectable fibrillin-1 or fibrillin-2 microfibrils. ADAMTS17-PCD and ADAMTS17-AD showed punctate deposition in the ECM with only minimal co-localization of ADAMTS17-PCD or ADAMTS17-AD with fibronectin (Fig. 9d), suggesting, together with protein-protein interaction data, that ADAMTS17 did not bind to FN or FN fibrils.

## Discussion

Determining the molecular mechanisms and substrates of orphan proteases, such as ADAMTS17, is crucial for understanding the pathogenesis of their associated genetic disorders. We have shown here that, i) ADAMTS17 undergoes extensive autoproteolysis, which occurs *in trans*, i.e. ADAMTS17 is its own substrate; ii) furin/PACE processing of ADAMTS17 is not required for autoproteolysis; iii) *O*-fucosylation of ADAMTS17 is required for its secretion; iv) ADAMTS17-PCD self-interacts via disulfide bond formation likely involving Cys201 in the propeptide; and, v) ADAMTS17 binds to fibrillin microfibrils, but does not cleave either of the fibrillin isoforms. These properties of ADAMTS17 identify it as a novel fibrillin binding protein and allow comparison of its properties with other ADAMTS proteins.

ADAMTS1, 4, and 5 require furin-mediated excision of their propeptides for proteolytic activity toward versican and aggrecan, but ADAMTS9 does not^32^,^44^,^45^,^46^,^47^,^48^. The ADAMTS13 propeptide is unusually short and structurally distinct harboring a single cysteine and the propeptide does not appear to regulate the enzymatic activity of ADAMTS13^49^,^50^. ADAMTS2, 3, and 14 have propeptides containing only two Cys residues^1^. Thus, ADAMTS17, along with ADAMTS9 and 13, is an unusual protease whose propeptide apparently lacks a role in zymogen latency. Rather, like ADAMTS9, the ADAMTS17 propeptide functions as an intramolecular chaperone, since its truncation prevents secretion^44^. Activation of matrix metalloproteases (MMPs) can be achieved by a cysteine switch^51^. Latency of MMPs is mediated by a free cysteine residue within the MMP propeptide that coordinates the zinc ion in the active site and blocks access to the substrate. MMP activation can be mediated by reducing agents or alkylation *in vitro*. A similar mechanism was also described for ADAM12, where either processing by furin or mutation of the free cysteine to alanine resulted in activation^52^. We identified a free cysteine residue (Cys^201^) in the ADAMTS17 propeptide. However, furin processing was not required for ADAMTS17 activation and the amino acid sequence context of Cys^201^ bears little resemblance to the consensus sequence described for MMPs^51^. Therefore, a cysteine switch mechanism similar to that in MMPs or some ADAMs is unlikely to mediate ADAMTS17 activation.

*O*-Fucosylation is a unique modification of TSR-containing proteins such as the ADAMTS proteins, all of which carry the required consensus sequence in one or more TSRs^53^. Suppression of either of the glycosyltransferases (POFUT2 or B3GLCT) acting sequentially in the *O*-fucosylation pathway resulted in impaired ADAMTS17 secretion, supporting its role in quality control during folding and secretion of TSR-containing proteins. Previously, it was shown that ADAMTS9, ADAMTS13, ADAMTSL1 and ADAMTSL2 were subjected to this quality control mechanism^34^,^35^,^54^,^55^. Interestingly, Peters Plus syndrome (PPS) (MIM #261540), which results from *B3GLCT* mutations is characterized by ocular anterior segment dysgenesis combined with short stature and brachydactyly, which are features of the WMS spectrum resulting from mutations in *ADAMTS10, ADAMTS17*, or *FBN1*^7^,^8^,^56^,^57^. PPS is thought to be a composite of phenotypes arising from impaired function of several B3GLCT target proteins as a result of their reduced secretion. On the basis of reduced ADAMTS17 secretion in the absence of B3GLCT and its ocular and skeletal expression and functions, ADAMTS17 could be one of the B3GLCT target proteins whose impaired secretion contributes to the PPS phenotype.

ADAMTS10, ADAMTS17, ADAMTSL2 and ADAMTSL4 have a robust genetic relationship with fibrillins^58^,^59^,^60^. ADAMTS10, whose mutations cause WMS, does not have an optimal furin processing consensus sequence and its zymogen is the predominantly secreted form^28^,^61^. Upon restoration of an optimal furin processing sequence by mutagenesis, ADAMTS10 cleaved recombinant fibrillin-1 *in vitro*^28^. In contrast, ADAMTS17 did not cleave fibrillin-1 or fibrillin-2, despite efficient processing by furin and autoproteolytic activity. Thus, ADAMTS10 and ADAMTS17 share the inability to cleave fibrillins, and ADAMTS10 behaves like ADAMTSL proteins, which constitutively lack proteolytic activity^62^,^63^. Unlike ADAMTS10, there was no evidence for ADAMTS17-mediated acceleration of microfibril formation. These distinct properties, as well as distinct expression profiles and intermolecular interactions may underlie some of the phenotypic differences observed between patients with *ADAMTS10* or *ADAMTS17* mutations, i.e. WMS and WMS-like syndrome, respectively^7^,^8^. ADAMTSL4, mutated in isolated ectopia lentis and ectopia lentis et pupillae, enhances fibrillin-1 assembly in cultured cells^29^. Strong expression of *Adamtsl4* by the embryonic and juvenile mouse lens epithelium and loss of the lens-zonule connection in *Adamtsl4* mutant mice suggests that ADAMTSL4 ensures anchorage of fibrillin microfibrils to the lens capsule^64^. ADAMTSL5 promoted assembly of fibrillin-1 microfibrils *in vitro*^65^, and ADAMTSL6 enhanced microfibril assembly upon transgenic overexpression in mice^30^. Neither is associated with a genetic condition in humans.

Despite the absence of binding to fibrillin-1 peptides, ADAMTS17 bound to fibrillin-1 containing microfibrils assembled by fibroblasts. One explanation could be that the ADAMTS17 binding site on fibrillin-1 microfibrils is contributed by two or more fibrillin-1 monomers and/or requires a specific spatial arrangement of fibrillin-1 within microfibrils rather than monomers in solution. In contrast, the apparent interference of ADAMTS17 with fibrillin-2 inclusion in microfibrils reflected suppression of *Fbn2* transcription.

The outcome of ADAMTS17 transcriptional suppression on fibrillin-2 assembly was akin to that ascribed to ADAMTSL2, which is mutated in geleophysic dysplasia, another short-stature-brachydactyly condition. ADAMTSL2 binds both fibrillin-1 and -2, and its inactivation in mice results in increased fibrillin-2 microfibrils in the bronchial wall in the lung without altering *Fbn2* gene expression, suggesting that ADAMTSL2 may participate in preferential inclusion of fibrillin-2 during microfibril assembly^27^,^66^. In contrast to ADAMTS17, ADAMTSL2 suppression of fibrillin-2 assembly did not involve transcriptional regulation. The mechanisms of transcriptional suppression of *Fbn2* by ADAMTS17 are unclear, but could involve signaling from disrupted ECM via integrins, direct binding of ADAMTS17 to a cell surface receptor, or disturbance of a feedback loop for fibrillin-2 expression. The profound effect of the ADAMTS17-AD ancillary domain construct on cell viability could be explained by similar mechanisms, such as outside-in signaling. However, since HEK293 cells stably express recombinant ADAMTS17-AD without apparent ill effect, a general cytotoxicity of ADAMTS17-AD is unlikely. ADAMTS17-AD could also interfere with the assembly or function of ECM networks other than fibrillin microfibrils and promote detachment and cell death (anoikis) of MEFs.

The expression of *ADAMTS17* mRNA was previously reported in the fetal brain, heart, lung, kidney and eye and in the adult brain, eye, and ovaries^7^,^67^. *ADAMTS17* mRNA was detected in the synovium and is expressed in normal human breast tissue and invasive ductal breast carcinoma^68^,^69^,^70^. Using a sensitive *in*-*situ* hybridization technique, we showed that *Adamts17* was expressed during mouse development in specific cell populations in a variety of tissues and was reduced after birth. This mimics the dynamic expression of *Fbn2*, which is predominantly expressed during embryonic development and downregulated after birth^24^,^25^. The data support a potential role for ADAMTS17 in the regulation of fibrillin-2 microfibril assembly or their functionalization. For example, during eye development, *Fbn2* is strongly expressed in the non-pigmented epithelium of the ciliary body, but not in the differentiating lens epithelium^39^. *Adamts17*, however, is expressed in the lens epithelium, but not the non-pigmented epithelium of the developing ciliary body. We speculate that ADAMTS17 could prevent the premature assembly of microfibrils in the vicinity of the lens and may play a role in the proper development of a ciliary zonule. An *Adamts17*-deficient mouse model is currently not available to test these possibilities *in vivo*.

In summary, we present the first biochemical and functional characterization of ADAMTS17 and expand the repertoire of ADAMTS/ADAMTSL proteins modulating the formation and function of fibrillin microfibrils. The findings expand the growing body of work suggesting that ADAMTS proteases constitute an important class of microfibril-associated regulatory proteins that are directly involved in microfibril formation or that confer tissue-specific functions to microfibrils in diverse ways^62^.

## Methods

All chemicals were purchased from Sigma-Aldrich (St. Louis, MO) or Thermo Fisher Scientific (Waltham, MA), unless specified. Cloning reagents such as restriction enzymes, T4 DNA ligase, and competent *E. coli* DH5α cells were purchased from New England Biolabs (Ipswich, MA).

### Cloning of ADAMTS17 Constructs

To generate a recombinant full-length, Myc/His-tagged human ADAMTS17 expression plasmid, a 1970 bp *Not*I × *Eco*RI fragment was excised from a synthetic DNA clone pENTR223.1-ADAMTS17 (NCBI RefSeq: XM_017021975, OriGene, Rockville, MD) and ligated into *Not*I × *Eco*RI digested pcDNA3.1/MycHis (-) A (Invitrogen, San Diego, CA), which was named pcDNA-TS17 Not_Eco. To add the 5′-end, a 432 bp PCR product was amplified from pENTR223.1-ADAMTS17 using the primers 5′-aactcgagaccatgtgtgacggcgccc-3′ (*Xho*I site underlined) and 5′-cccgagtagaagcacagctc-3′ and subcloned into the pGEM-T Easy vector (Promega, Madison, WI). The *Xho*I × *Not*I fragment was then isolated and ligated into the *Xho*I × *Not*I digested pcDNA-TS17 Not_Eco plasmid resulting in the plasmid pcDNA-TS17 Not_Eco-Nterm. Similarly, a 1013 bp fragment of the ADAMTS17 3′-end was PCR amplified from pENTR223.1-ADAMTS17 with the primers 5′-ccgctgcacttgatggtgttgttatt-3′ and 5′-ttggatcccgagttcggcggtggct-3′ (*Bam*HI site underlined), subcloned in pGEM-T Easy, and the *Eco*RI × *Bam*HI fragment was ligated into *Eco*RI × *Bam*HI digested pcDNA-Ts17 Not_Eco-Nterm. This resulted in the final expression plasmid pcDNA-ADAMTS17, encoding full-length human ADAMTS17 with the endogenous signal peptide, and a C-terminal Myc/His_6_-tag. An N-terminal ADAMTS17 construct comprising the ADAMTS17 propeptide, the catalytic domain and the disintegrin-like domain (ADAMTS17-PCD) (Fig. 1a) was generated by introducing a unique *Bam*HI restriction site into the full-length pcDNA-ADAMTS17 plasmid at nucleotide position 1709 to 1714 of human ADAMTS17. Site-directed mutagenesis was performed with the QuikChange XL site-directed mutagenesis kit (Agilent Technologies, Santa Clara, CA) using the primer pair 5′-gagcatgtggacggatcctggagcccgtggg-3′ and 5′-cccacgggctccaggatccgtccacatgctc-3′ (mutated nucleotides underlined) and with pcDNA-ADAMTS17 plasmid as PCR template. The plasmid containing the introduced mutations was digested with *Bam*HI and the gel-purified 7104 bp fragment re-ligated, resulting in the plasmid pcDNA-ADAMTS17-PCD. To clone the ancillary domain extending from TSR1 to the PLAC module (Fig. 1a), a 1668 bp PCR product was amplified using the primer pair 5′-ataagctttggagcccgtggggcgcc-3′ (*Hind*III site underlined) and 5′- atggatccggcgagttcggcggtggctgg-3′ (*Bam*HI site underlined) with pcDNA-ADAMTS17 as template and subcloned into the pGEM-T Easy vector (Promega, Madison, WI). The 1656 bp *Hind*III × *Bam*HI fragment was ligated into *Hind*III × *Bam*HI restricted pSecTag 2B vector. The resulting plasmid, pSecTag-ADAMTS17-AD, contains an Igκ-chain leader sequence followed by the ADAMTS17 ancillary domain and a C-terminal Myc/His_6_-tag. To clone ADAMTS17 1C (TSR1-cysteine rich domain) and 25P (TSR2-PLAC domain) (Fig. 3a), we generated PCR products using the primer pairs 5′-ataagctttggagcccgtggggcgcc-3′/5′-atctcgagcgcaggtcttgccgtcccc-3′ (1C) and 5′-ataagctttggacccacagcggctgg-3′/5′-atctcgagccgagttcggcggtggctg-3′ (25P) (*Hind*III and *Xho*I sites underlined, respectively) and pSecTag-ADAMTS17-AD as template DNA. The PCR products were digested with *Hind*III × *Xho*I and ligated into *Hind*III × *Xho*I digested pSecTag 2B resulting in the plasmids pSecTag-ADAMTS17-1C and pSecTag-ADAMTS17-25P, respectively. To clone ADAMTS17-2 (starting at second furin processing site and ends after disintegrin-like domain), ADAMTS17-Furin (catalytic domain – disintegrin-like domain), and ADAMTS17-Cat (catalytic domain) (Fig. 4g), we generated PCR products with the following primers: 5′-ataggatcccccgccgagctgtgcttc-3′ and 5′-atctcgagcgtctccgtccacatgctcc-3′ for ADAMTS17-2, 5′-ataggatccaacgctatccggctcacc-3′ and 5′-atctcgagcgtctccgtccacatgctcc-3′ for ADAMTS17-Furin, and 5′-ataggatccaacgctatccggctcacc-3′ and 5′-atctcgagcgcaggtgctgacttttgac-3′ for ADAMTS17-Cat, respectively (*Xho*I and *Bam*HI sites are underlined). The PCR products were digested with *Xho*I × *Bam*HI and ligated into the *Xho*I × *Bam*HI digested pSecTag 2B plasmid, resulting in the expression plasmids pSecTag-ADAMTS17-2, pSecTag-ADAMTS17-Furin and pSecTag-ADAMTS17-Cat, respectively. To introduce the Glu^390^ to Ala (EA) active site mutation in pcDNA-ADAMTS17 and pcDNA-ADAMTS17-PCD, a GCC codon (Ala) replacing the wild-type GAG codon (Glu) was introduced in a 371 bp gBlock DNA fragment (Integrated DNA Technologies, Coralville, IA) spanning nucleotide 1100 to nucleotide 1470 of ADAMTS17. The gBlock DNA and pcDNA-ADAMTS17 or pcDNA-ADAMTS17-PCD were digested with *Psh*AI × *Xcm*I, gel purified and ligated using the NEBuilder® HiFi DNA Assembly Kit resulting in pcDNA-ADAMTS17^EA^ and pcDNA-ADAMTS17-PCD^EA^, respectively. To introduce the Arg^223^ to Ala (RA) furin site mutation, two overlapping PCR products were assembled together with *Not*I × *Bsr*GI digested ADAMTS17-PCD using the NEBuilder® HiFi DNA Assembly Kit (New England Biolabs, Ipswich, MA). The PCR products were generated with the primer pairs 5′- cttcgaggtggaggaggc-3′/5′- ggatagcgttcgcccgctcccgcca-3′ and 5′-tggcgggagcgggcgaacgctatcc-3′/5′-cagcgcaagatgagtggtcat-3′ (mutated nucleotides underlined) using ADAMTS17-PCD as template DNA. The *Not*I and *BsrG*I fragment from pcDNA-ADAMTS17-PCD^RA^ was then cloned into pcDNA-ADAMTS17, resulting in pcDNA-ADAMTS17^RA^. All plasmid sequences were verified by DNA sequencing (Cleveland Clinic Lerner Research Institute Genomics Core). Plasmid DNA for transfections was purified with QIAprep Miniprep or Qiagen Plasmid Midi Kit (Qiagen Corp, Valencia, CA).

### Cell Culture and Transfection

Human embryonic kidney cells (HEK293F, HEK293T) and monkey kidney cells (COS-1) were purchased from ATCC (Manassas, VA) and maintained in DMEM supplemented with 10% FBS, 100 units/ml penicillin, 100 μg/ml streptomycin in a 5% CO_2_ atmosphere in a humidified incubator at 37 °C. HEK293F cells were seeded in 12-well plates and transfected with 1–2 μg plasmid DNA using Lipofectamine 3000 according to the manufacturer’s protocol. Stable clones were selected with geneticin (G418) for pcDNA based plasmids or zeocin (Invitrogen, San Diego, CA) for pSecTag 2B based plasmids and were analyzed for protein expression and secretion by western blotting. For *O*-fucosylation analysis, HEK293T cells were cultured in DMEM supplemented with 10% bovine calf serum in 6 cm cell culture dishes. 0.5–1 μg plasmid DNA was transfected using polyethylenimine (PEI).

### Protein Expression and Western Blotting

Stable expressing cells or transiently transfected cells were analyzed for the presence of recombinant proteins in conditioned medium and the cell layer. Confluent cells were cultured for 48–72 h after transfection in serum-free DMEM medium in a 12-well tissue culture plate (BD Bioscience, San Jose, CA). For *O*-fucosylation analysis, transiently transfected HEK293T cells were cultured for 48 h in reduced serum medium (OptiMEM). Conditioned medium was harvested, cell debris removed by centrifugation, and equal volumes subjected to SDS-PAGE under reducing conditions. The cell layer was washed once with 1 ml PBS (137 mM NaCl, 2.7 mM KCl, 10 mM Na_2_KPO_4_, 1.8 mM KH_2_PO_4_) and lysed in 0.1% NP40, 0.01% SDS, 0.05% Na-deoxycholate in PBS (RIPA buffer). Cell lysates were incubated for 5 min at 4 °C with rotation end-over-end. The lysate was cleared by centrifugation (5 min, >20,000 g, 4 °C) and equal volumes were subjected to SDS-PAGE under reducing conditions. Proteins were transferred onto PVDF membranes (Immobilon P for enhanced chemiluminescence, Immobilon F for infrared fluorescent detection, EMD Millipore, Billerica, MA) for 1.5 h at 70 V at 4 °C in 25 mM Tris, 192 mM glycine, 20% methanol buffer. The membrane was blocked with 5% (w/v) milk in TBS (10 mM Tris-HCl, pH 7.2, 0.15 mM NaCl) for 1 h at room temperature and primary antibodies were diluted in 5% (w/v) milk in TBS + 0.1% Tween 20 (TBST) and incubated overnight at 4 °C. The following antibodies and dilutions were used: anti-Myc (9E10, Invitrogen, 1:500–1:1,000), mouse monoclonal antibody to human ADAMTS17 (Abcam, Cambridge, MA; ab58099, 1:500), rabbit polyclonal antibody to human ADAMTS17 (N-term) (Abgent, San Diego, CA; 1:500), rabbit polyclonal antibody to human ADAMTS17 (catalytic domain) (Abcam, ab59828, 1:750). The polyclonal antibodies against human fibrillin-1 (C-terminus) (1:1,000) and fibrillin-2 (N- and C-terminus) (1:500) were described previously^42^,^71^,^72^. Membranes were washed three times with TBST for 5 min at RT and incubated with the corresponding secondary horseradish peroxidase coupled goat-anti-mouse or goat-anti-rabbit antibodies (Jackson ImmunoResearch Laboratories, West Grove, PA; 1:2,500 in blocking buffer for enhanced chemiluminescence) in 5% milk in TBST or fluorophore-labeled IRDye goat-anti-mouse or goat-anti-rabbit secondary antibodies (LI-COR Biosciences, Lincoln, NE) in 5% milk in TBST + 0.01% SDS for 1 h at room temperature. For enhanced chemiluminescence, membranes were washed three times with TBST for 5 min at room temperature and the bound antibodies were visualized using enhanced chemiluminescence (ECL Prime, GE Healthcare, Piscataway, NJ, USA). For infrared detection, blots were washed three times with TBST for 5 min, one time with TBS for 5 min at room temperature, and were scanned wet on an Odyssey CLx scanner (LI-COR Biosciences, Lincoln, NE).

### Protein Purification

Recombinant ADAMTS17-PCD and ADAMTS17-AD were purified by Ni^2+^-NTA affinity chromatography using an Äkta purifier chromatography system (GE Healthcare, Piscataway, NJ, USA). 1–2 l of conditioned medium was concentrated by ultrafiltration through a 30-kDa cutoff membrane (EMD Millipore, Billerica, MA) at 4 °C. The concentrate was dialyzed twice against 1 l chromatography buffer (20 mM HEPES, 500 mM NaCl, pH 7.4) at 4 °C and loaded on a 1 ml Ni^2+^-NTA column (GE Healthcare, Piscataway, NJ, USA). Bound protein was eluted with a linear imidazole gradient (40 ml, 0–500 mM in 20 mM HEPES, 500 mM NaCl, pH 7.4). 1 ml fractions were collected and analyzed for the presence of ADAMTS17-PCD or ADAMTS17-AD protein by SDS-PAGE followed by Coomassie Brilliant Blue staining. Fractions containing purified ADAMTS17-PCD or ADAMTS17-AD were pooled, concentrated (if necessary), dialyzed against PBS, and stored at −80 °C.

### Identification of semi-tryptic peptides

Proteins were acetone-precipitated from conditioned medium containing recombinant ADAMTS17 or ADAMTS17^EA^, solubilized in 6 M urea, disulfide-bonds were reduced with 200 mM dithiothreitol (DTT) and free cysteines alkylated with 200 mM iodoacetamide. The urea concentration was diluted to below 2 M and samples were trypsin digested overnight. The trypsin digests were subjected to a C18 cleanup step, evaporated to <10 μL in a Speedvac and resuspended in 1% acetic acid in a final volume of ~50 μL for liquid-chromatography coupled mass-spectrometry (LC-MS/MS) analysis, using a Finnigan LTQ-Orbitrap Elite hybrid mass spectrometer system equipped with a Dionex 15 cm x 75 μm Acclaim Pepmap C18 reversed phase capillary chromatography column (Thermo Scientific, Sunnyvale, CA). The identification of semi-tryptic ADAMTS17 peptides was performed by searching the MS/MS spectra specifically against the sequence of ADAMTS17 using the program Sequest (bundled into Proteome Discoverer v. 1.4.0) considering the formation of semi-tryptic peptides. Additional search criteria include a 10 ppm and 0.6 Da mass error for MS and MS/MS scans respectively, oxidation of methionine as a dynamic modification, and carbamidomethylation of cysteines as a static modification. All semi-tryptic ADAMTS17 peptides were high confidence identifications and were subjected to manual validation. The relative abundance of these semi-tryptic peptides was determined by plotting chromatograms for both the semi-tryptic and fully tryptic forms of each peptide, integrating these chromatographic peaks, and using the resulting peak areas to determine the peak area ratio: semi-tryptic/fully tryptic.

### Identification of solvent-accessible cysteine residues

30 μg purified ADAMTS17-PCD in Tris-HCl buffer containing 6 M urea was treated with 200 mM N-ethylmaleimide (NEM) to label solvent accessible free cysteinyl residues, then reduced with 200 mM DTT, and alkylated with 200 mM iodoacetamide prior to protease digestion. Mass-spectrometry grade trypsin, chymotrypsin, and GluC (Roche Diagnostics, Indianapolis, IN) were added at a 1:20 (protease to protein) ratio, and the digest was incubated overnight at room temperature for completion. The protein digest was desalted using C18 SPE method (PepClean C-18 spin column) and reconstituted in 30 μL 1% acetic acid for LC-MS/MS analysis as described above. The identification of cysteine-containing ADAMTS17 peptides was performed by searching the MS/MS spectra specifically against the sequence of ADAMTS17 using the program Sequest (bundled into Proteome Discoverer v. 1.4.0) considering the formation of both carbamidomethylated and NEM modified cysteine residues. All cysteine-containing peptides were high confidant identifications and were subjected to manual validation.

### Analysis of N-glycosylation and *O*-fucosylation

ADAMTS17^EA^ in conditioned medium from transiently transfected HEK293F cells was deglycosylated with PNGaseF or Endoglycosidase H (New England Biolabs, Ipswich, MA) according to the manufacturer’s protocol. Digested proteins were analyzed by western blotting as described above. For analysis of protein *O*-fucosylation, ADAMTS17 constructs 1C and 25P were transiently expressed in HEK293T cells and proteins were purified from conditioned medium using Ni^2+^-NTA chelating chromatography resin (Qiagen, Hilden, Germany). The purified proteins were reduced, alkylated, subjected to in-gel digestion with trypsin or chymotrypsin (Promega, Madison, WI), and the resulting peptides were analyzed on an Agilent 6340 HPLC-Chip Cube nano LC-Ion trap mass spectrometer as described previously (37). *O*-Fucosylated peptides were identified by neutral loss searches and semi-quantitative Extracted Ion Chromatograms (EIC) of selected ions were generated to compare relative amounts of relevant glycoforms of each peptide. EIC chromatograms were smoothed using a Gaussian algorithm (37). To determine the impact of *O*-fucosylation on ADAMTS17 secretion, we generated *POFUT2* and *B3GLCT* knockouts in HEK293T cells using CRISPR/Cas9 methodologies. The generation of the *POFUT2* knockout cell line was described recently^34^. The same protocol was used to generate the *B3GLCT* knockouts except that we used three *B3GLCT*-specific guide RNAs (gRNA-1: 5′-GGATGCGGCCGCCCGCCTGC-3′; gRNA-2: 5′-TTCTGAAGATACAAAGAAAG -3′; gRNA-3: 5′-ATACAGGATTTGGAGAAAAG -3′), and corresponding primer pairs to confirm genomic targeting (primers for gRNA-1: 5′-GGCAGACGCTGGAAGC-3′ and 5′-CTACTTACCCAGGGAGCAGG-3′; primers for gRNA-2: 5′- AGCTGCTTAAAAATGAGCAAAA -3′ and 5′-AAAACCAAGTGGATCAGCCT-3′; primers for gRNA-3: 5′-TGGTCCCTTAGGTTTCGGTC-3′ and 5′-ACATAAATCCACCACATGCCA-3′). Loss of B3GLCT protein was confirmed by western blotting of cell lysates with anti-human B3GLCT antibody (OriGene, catalog no. TA316142, Rockville, MD). Secretion assays using ADAMTS17 constructs compared control HEK293T cells to POFUT2 or B3GLCT knockout HEK293T cells, using co-transfected human IgG as a secretion control, as recently described^34^. The conditioned media and cell lysates were analyzed by reducing SDS-PAGE followed by western blotting with anti-myc and anti-human IgG (Rockland, Limerick, PA), and the blots were quantified using the Odyssey CLx scanner.

### Cross-linking experiments

10 μg purified non-reduced or reduced and alkylated ADAMTS17-PCD was incubated with different amounts of bis(sulfosuccinimidyl)suberate (BS^3^), an amine-to-amine cross linker with a 11.4 Å spacer for 30 min at RT. The reaction was terminated by quenching with 100 mM Tris-HCl, pH 7.5 and samples were separated on a 7.5% reducing SDS-PAGE followed by Coomassie Brilliant Blue staining.

### RNA *In*-*situ* Hybridization

16.5 day old mouse embryos were fixed in 4% paraformaldehyde overnight at 4 °C, processed and paraffin-embedded. Fresh 6 μm sections were used for *in*-*situ* hybridization using RNAscope (Advanced Cell Diagnostics, Newark, CA) following the manufacturer’s protocol. All steps requiring incubation at 40 °C were performed in the HybEZ Oven (Advanced Cell Diagnostics, Newark, CA). Tissue localization of the specific probe against mouse *Adamts17* mRNA (#316441) was detected with the RNAscope 2.0 HD detection kit “RED”. Probes against peptidylprolyl isomerase B (Cyclophylin B, *Ppib*) or bacterial dihydrodipicolinate reductase (*DapB*) mRNA (#313911, #310043) were used as positive and negative control, respectively. Sections were counterstained with hematoxylin prior to coverslipping with Cytoseal 60 (Electron Microscopy Science, Hatfield, PA, USA).

### Protein-protein interaction analysis by surface plasmon resonance (SPR)

Purified ADAMTS17-PCD and ADAMTS17-AD were diluted in 10 mM sodium acetate pH 4.5 and immobilized on a BIAcore CM5 sensor chip (GE Healthcare, Piscataway, NJ, USA) with the amine coupling kit according to the manufacturer’s instructions. For analysis in a BIAcore 3000 instrument (GE Healthcare, Piscataway, NJ, USA), 3300 resonance units for ADAMTS17-PCD and 2530 resonance units for ADAMTS17-AD were coupled to the chip. Kinetic analyses were performed at 25 °C in TBS including 2 mM CaCl_2_ and 0.005% SP20 (running buffer) at a flow rate of 20 μl/min. Purified recombinant fibrillin-1 (FBN1-N, FBN1-C) and fibrillin-2 (FBN2-N, FBN2-C) peptides were described previously^42^,^73^ and were diluted in running buffer at the respective concentrations and injected in series through an uncoupled control flow cell followed by the flow cell with immobilized ADAMTS17-PCD or ADAMTS17-AD. Association was allowed for 3 min followed by a 5 min dissociation phase. To analyze calcium dependency of fibrillin binding to ADAMTS17-PCD or ADAMTS17-AD, FBN2-N and FBN2-C were diluted in TBS including 10 mM EDTA and 0.005% SP20 and the same buffer was used as running buffer. The CM5 chip was regenerated by injecting 60 μl of 500 mM EDTA, pH 8.6 before analyzing a different peptide. The kinetic data were calculated using BIAevaluation 4.0.1 software (GE Healthcare, Piscataway, NJ, USA) assuming a 1:1 stoichiometry.

### Digestion of FBN1 and FBN2 with ADAMTS17-PCD

5 μg of purified FBN1 or FBN2 peptides were incubated with purified ADAMTS17-PCD in PBS for 5 h at 37 °C. Samples were separated by 7.5% SDS-PAGE under reducing conditions and the gel was stained with Coomassie Brilliant Blue.

### ADAMTS17 microfibril binding using cultured fibroblasts

Wild-type (WT) mouse embryonic fibroblasts (MEF, 50000–75000 cells/well), human dermal fibroblasts (HDF) derived from foreskin explants, or rat aortic smooth muscle cells A7r5 (ATCC, Manassas, VA) were cultured in the presence of 50 μg purified ADAMTS17-PCD, ADAMTS17-AD, or PBS buffer control in 8-well chamber slides (BD Bioscience, San Jose, CA). Recombinant ADAMTS17-PCD or ADAMTS17-AD was added 24 h after seeding the cells and cells were cultured for up to five additional days. Cells were fixed in ice-cold 70% methanol/30% acetone for 5 min at room temperature. After blocking with 10% NGS in PBS (blocking buffer) for 1 h at room temperature, cells were incubated with the following antibody combinations, diluted in blocking buffer, for 2 h at room temperature: anti-Myc (1:300) + anti-fibrillin-1-C (1:500), anti-Myc (1:300) + anti-fibrillin-2-N (1:300), or anti-Myc (1:300) + anti-fibronectin (pAB 2033, 1:500, EMD Millipore, Billerica, MA)^71^,^72^. Cells were washed three times with PBS for 5 min at room temperature and incubated with the corresponding Alexa-Fluor labeled secondary goat-anti-mouse or goat-anti-rabbit antibodies (diluted 1:350 in blocking buffer, Jackson ImmunoResearch Laboratories, West Grove, PA) for 1.5 h at room temperature. Cells were washed three times with PBS for 5 min at room temperature and mounted with ProLong Gold Antifade Reagent with DAPI. Slides were imaged using an Olympus BX51 upright fluorescent microscope equipped with a CCD camera (Leica Microsystems). Post-acquisition image analysis and counting of cell nuclei was performed with Corel PHOTO-PAINT or ImageJ (National Institutes of Health, Bethesda, MD)^74^.

### RNA isolation and quantitative real-time PCR (qRT-PCR)

MEFs isolated from WT mice were seeded in 12-well plates (150,000/well) and after cell attachment, 150 μg purified ADAMTS17-PCD or an equivalent volume of PBS in complete DMEM was added. Cells were incubated for 72 h and total RNA was extracted using TRIzol (Ambion, Thermo Fisher Scientific, Waltham, MA) according to the manufacturer’s protocol. RNA concentration was determined using a ND-1000 spectrophotometer and 1 μg of total RNA was reverse transcribed using the High-Capacity cDNA Reverse Transcription Kit (Applied Biosystems, Foster City, CA) according to the manufacturer’s protocol. Quantitative real-time PCR was performed in duplicates in 96-well hard-shell PCR plates (Bio-Rad, Hercules, CA) using 0.125 μl cDNA and Bullseye EvaGreen qPCR Mix (MIDSCI, St. Louis, MO) in a total volume of 10 μl in a CFX96 Real-Time System (Bio-Rad, Hercules, CA). The primers for mouse *Fbn1, Fbn2, FN*, and *Gapdh* were described recently^27^. The following primer pairs were used to detected human gene expression: *FBN1* (5′-accctatgccaagttgatcc-3′/5′-actgacacttgaatgacccc-3′), *FBN2* (5′-gagcggtgtgaactagatacag-3′/5′-gcactcgcaatgaaaagatcc-3′), *FN* (5′-actgtacatgcttcggtcag/5′-agtctctgaatcctggcattg-3′), and *GAPDH* (5′-acatcgctcagacaccatg-3′/5′-tgtagttgaggtcaatgaaggg-3′).

## Additional Information

**How to cite this article**: Hubmacher, D. *et al*. Unusual life cycle and impact on microfibril assembly of ADAMTS17, a secreted metalloprotease mutated in genetic eye disease. *Sci. Rep.* **7**, 41871; doi: 10.1038/srep41871 (2017).

**Publisher's note:** Springer Nature remains neutral with regard to jurisdictional claims in published maps and institutional affiliations.

## Supplementary Material

Supplemental Information
