# Supplementary material for: Unusual life cycle and impact on microfibril assembly of ADAMTS17, a secreted metalloprotease mutated in genetic eye disease
Source: Sci Rep. 2017 Feb 8;7:41871. doi: 10.1038/srep41871 (PMC5296908; doi:10.1038/srep41871)
Supplement: Supplemental Information [file srep41871-s1.pdf]

Unusual life cycle and impact on microfibril assembly of ADAMTS17, a secreted metalloprotease mutated in genetic eye disease

Dirk Hubmacher[1,*], Michael Schneider[2], Steven J. Berardinelli[3], Hideyuki Takeuchi[3], Belinda Willard[4], Dieter P. Reinhardt[5], Robert S. Haltiwanger[2,3] and Suneel S. Apte[1,*]

Supplemental Information:

Supplemental Figure 1: Mass spectra for autoproteolysis of ADAMTS17. Culture medium from HEK cells expressing wild-type ADAMTS17 and ADAMTS17^{EA} was analyzed by mass spectrometry. Predicted b and y ions are indicated. For peptide list see Figure 1E and Supplemental Table 1.

Supplemental Figure 2: Mass spectra of *O*-fucosylation on ADAMTS17. ADAMTS17 fragments were purified from HEK293T cells and prepared for mass spectrometry as described in Materials and Methods. Peptides corresponding to TSRs with fucose modification sites were identified and are indicated above each spectrum (see also Supplemental Table 2). The top panel for each peptide shows the MS spectra at a specific retention time. Red diamonds indicate ions chosen for fragmentation. Bottom panels represent the MS2 spectra corresponding to the appropriate parent ion. Blue diamonds in the MS2 spectra indicate the position of the parent ion. Peaks representing different glycoforms of each peptide and predicted b and y ions are indicated. Tables to the right of the spectra show ions searched in the EICs in Fig. 3D. Red triangles represent fucose (dHexose). Blue circles are glucose (Hexose).

Supplemental Figure 3: Mass spectra for BS^3 cross-linking of ADAMTS17-PCD. Purified ADAMTS17-PCD was incubated with BS^3 cross-linker and cross-linked peptides were analyzed by mass spectrometry. Predicted b and y ions are indicated. For peptide list see Supplemental Table 3.

Supplemental Figure 4: Mass spectra for NEM and IAA modified ADAMTS17-PCD. Purified ADAMTS17-PCD was incubated with NEM and IAA and analyzed by mass spectrometry. Predicted b and y ions are indicated. For peptide list see Supplemental Table 4.

Supplemental Figure 5: Full-length western blots shown in Fig. 3. (a) Full-length western blot showing for the N-glycosylation analysis of ADAMTS17-EA. Note the BSA band due to the presence of 10% bovine serum albumin in the conditioned medium. The signal is not caused by reacting with the antibody, but by the presence of high amounts of protein. **(b)** Full-length western blot for the lysates from the analysis of the role of *O*-fucosylation in the secretion of ADAMTS17 peptides. The blots probed with anti-myc antibody are shown in red and the IgG controls in green. Lanes used in Fig. 3e are outline with a white rectangle. **(C)** Full-length western blot for the media from the analysis of the role of *O*-fucosylation in the secretion of ADAMTS17 peptides. The blots probed with anti-myc antibody are shown in red and the IgG controls in green. Lanes used in Fig. 3e are outline with a white rectangle. Two different exposures are shown. The low exposure blots were used to detect the 1C and 25P peptides and the high exposure blot was used for the ADAMTS17-EA protein.

Supplemental Table 1: Peptides targeted in LC-MS/MS analysis of ADAMTS17 autoproteolysis.

Supplemental Table 2: Peptides from the MS analysis of ADAMTS17 *O*-fucosylation.

Supplemental Table 3: Peptides from the LC-MS/MS analysis of the tryptic digest of cross-linked ADAMTS17-PCD.

Supplemental Table 4: Peptides used for selective reaction monitoring analysis of NEM- accessible cysteine residues.

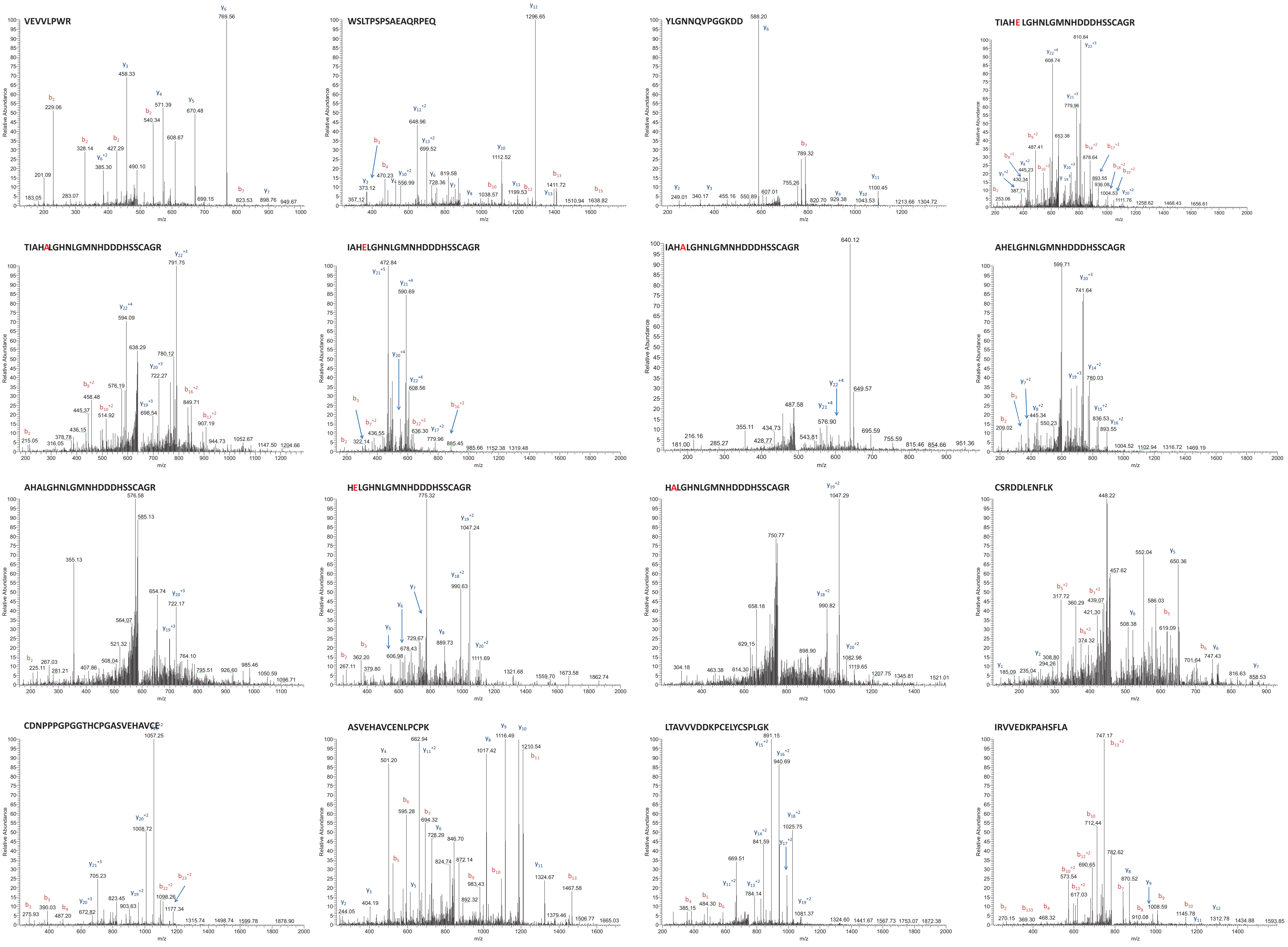

Supplemental Figure 1, Hubmacher et al.

TSR1

555CSRTCGTGARF565

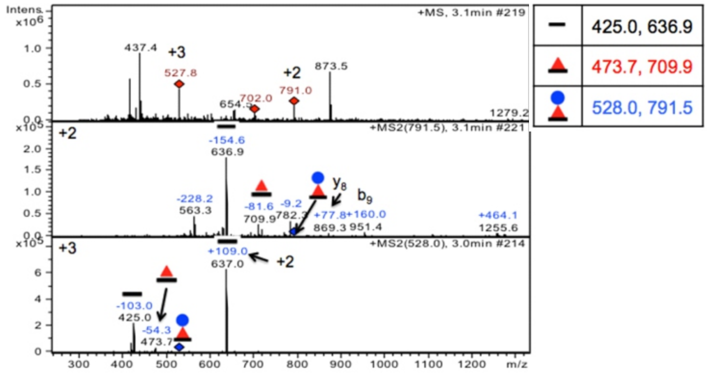

TSR3

871SPCSATCEKGF881

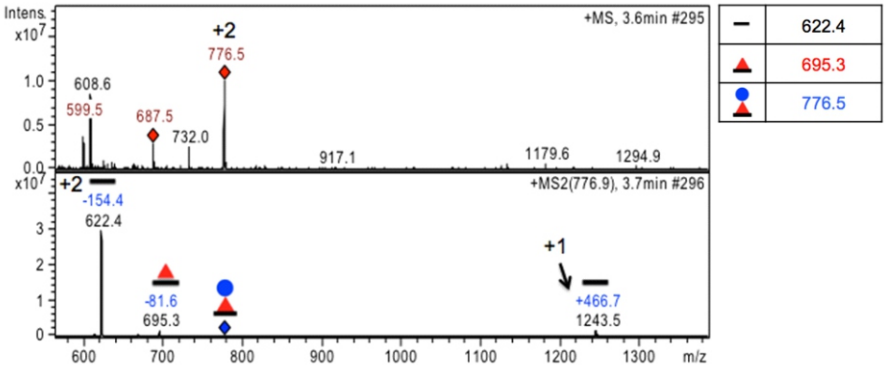

TSR4

931SQCSASCGKGVW942

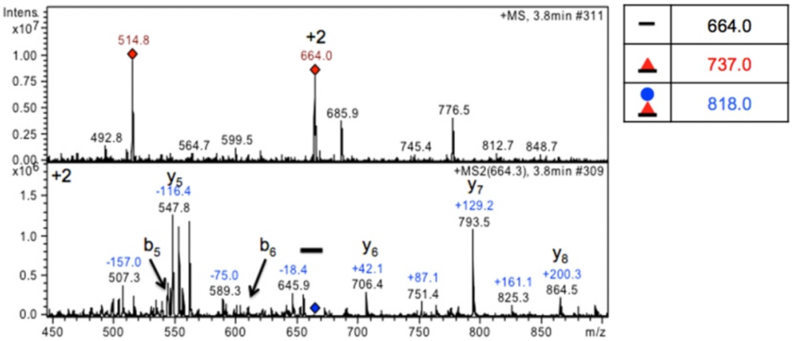

TSR3

871SPCSATCEKGFQHREVTCVY889

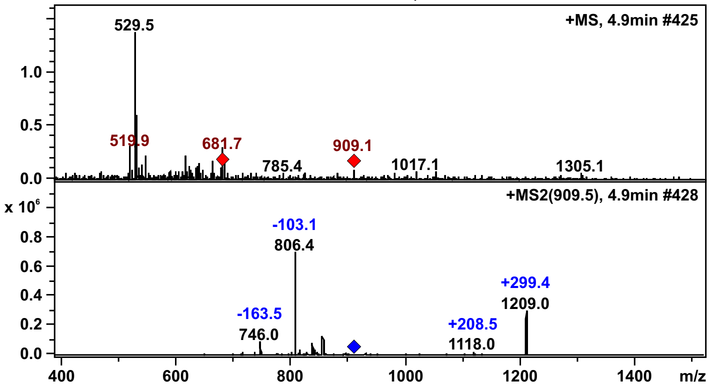

TSR5

978TGDWSTCSSTCGK990

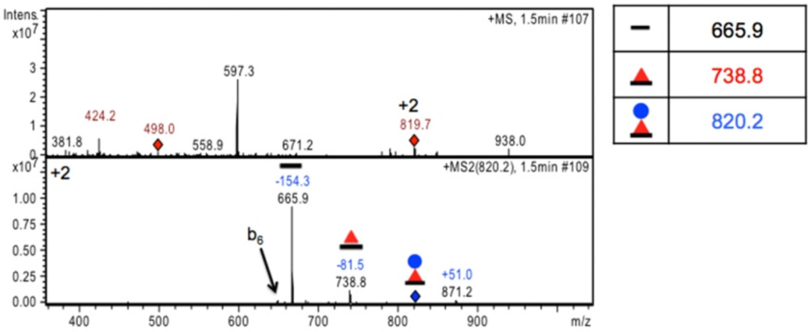

Supplemental Figure 2, Hubmacher et al.

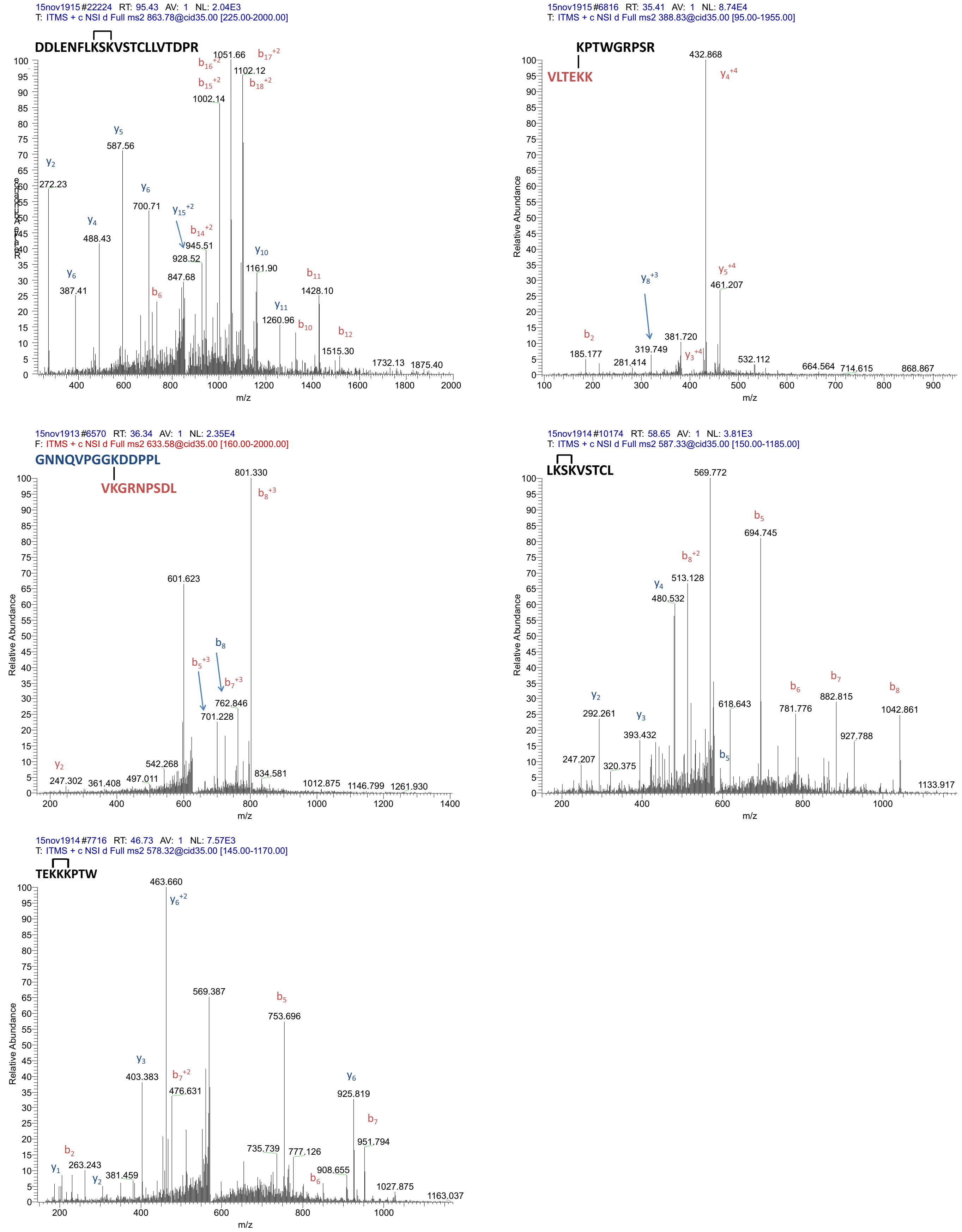

Supplemental Figure 3, Hubmacher et al.

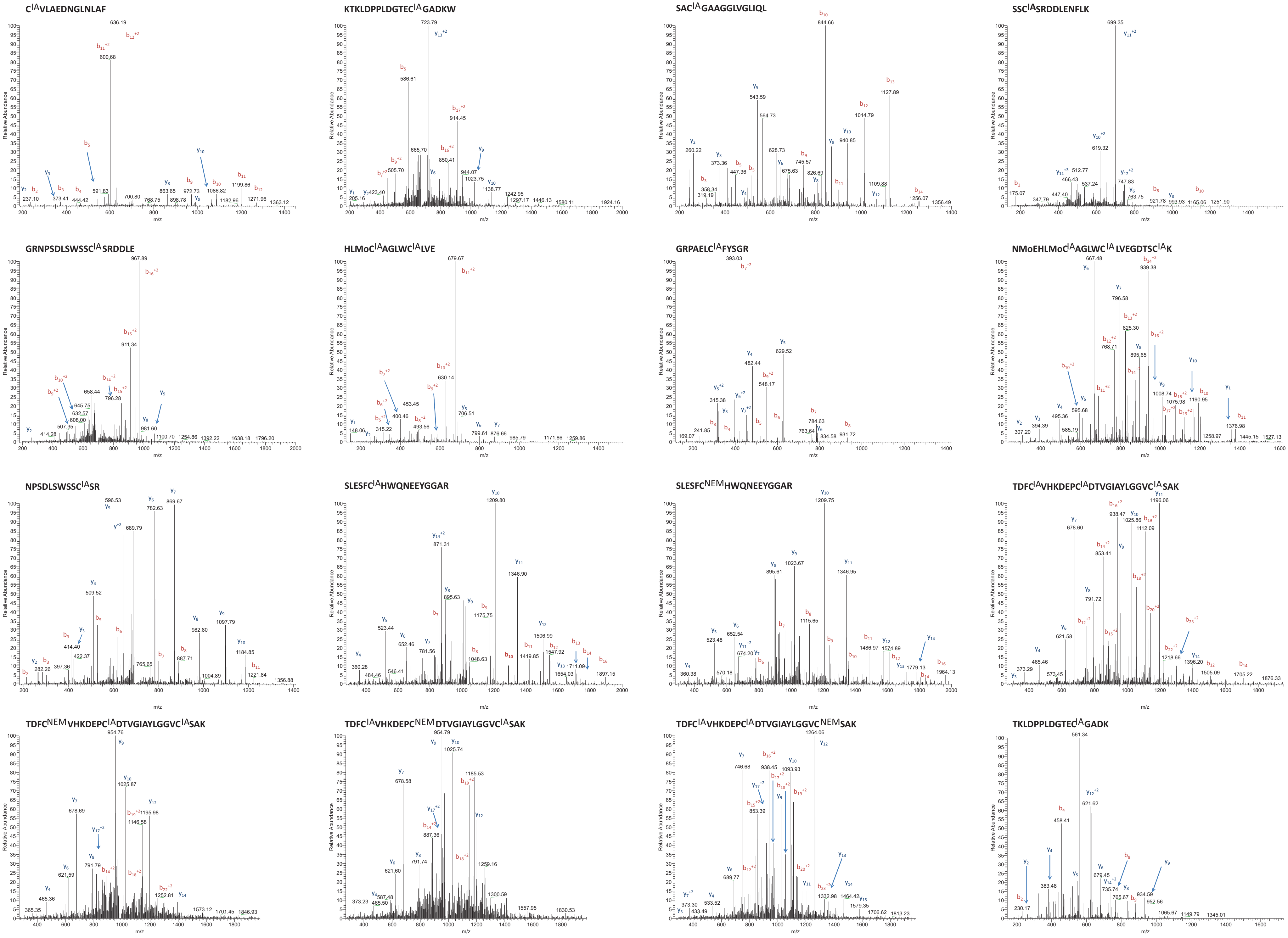

Supplemental Figure 4, Hubmacher et al.

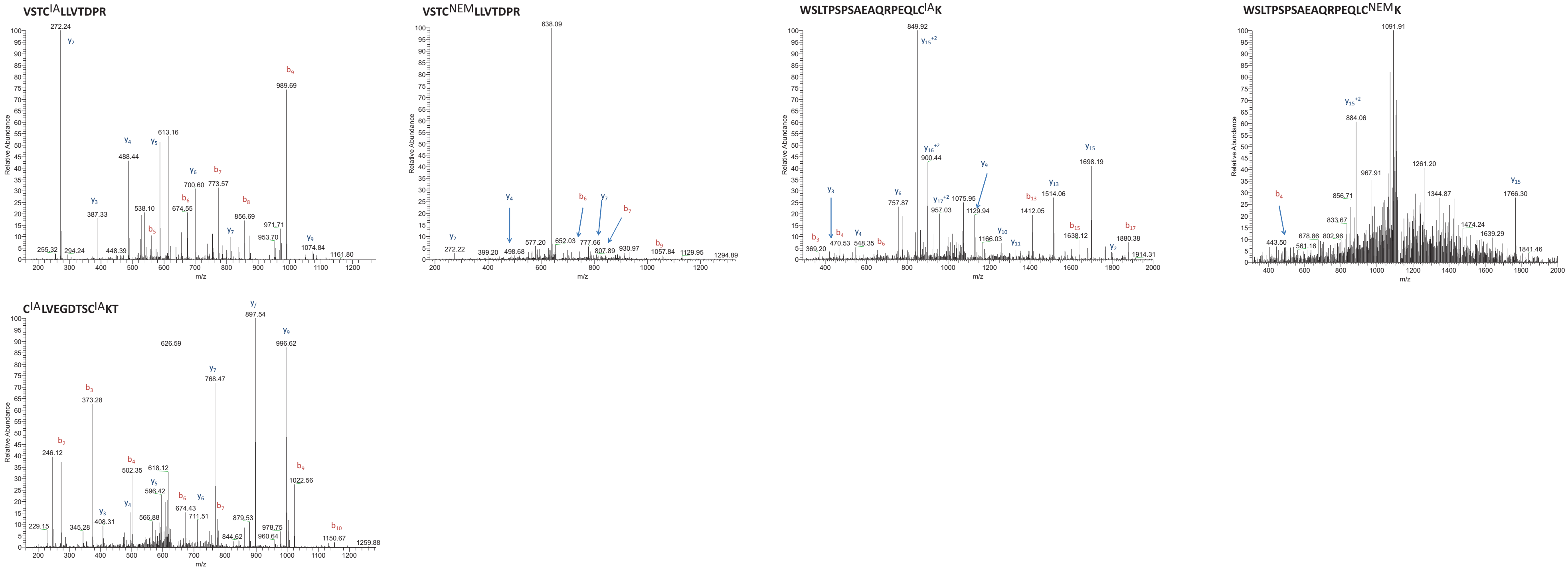

Supplemental Figure 4 contd., Hubmacher et al.

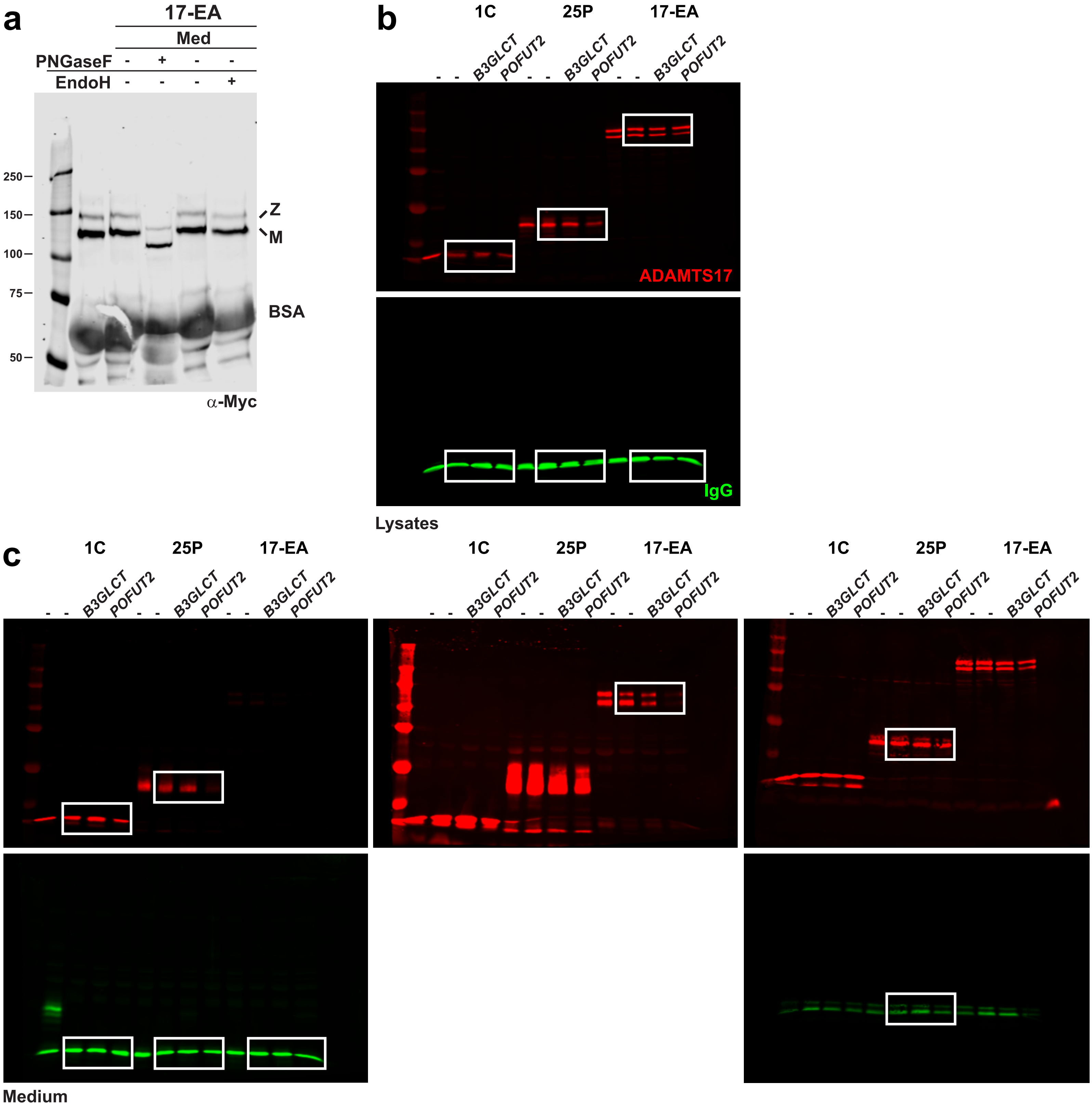

Supplemental Figure 5, Hubmacher et al.

Supplemental Table 1: Peptides targeted in LC-MS/MS analysis of ADAMTS17 autoproteolysis[1].

Sequence	z (n)	[M+nH] (Da)	M/z (Da)	WT	EA	WT/EA
^{35}VEVVLPWR^{42}	2	997.60	499.3	0.003	0.002	1.5
^{184}WSLTPSPSAEAQRPEQ^{196}	2	1783.9	892.4	0.011	0.002	5.5
^{320}YLGNNQVPGGKDD^{332}	2	1376.6	688.8	0.031	0.046	0.7
^{386}TIAHELGHNLGMNHDDDHSSCAGR^{409}	4	2644.1	661.8	0.210	-	3.6
^{386}TIAHALGHNLGMNHDDDHSSCAGR^{409}	4	2586.1	647.3	-	0.058	-
^{387}IAHELGHNLGMNHDDDHSSCAGR^{409}	5	2543.1	509.4	0.110	-	6.5
^{387}IAHALGHNLGMNHDDDHSSCAGR^{409}	5	2484.1	497.8	-	0.017	-
^{388}AHELGHNLGMNHDDDHSSCAGR^{409}	4	2430.0	608.3	0.041	-	2.9
^{388}AHALGHNLGMNHDDDHSSCAGR^{409}	4	2371.9	593.8	-	0.014	-
^{389}HELGHNLGMNHDDDHSSCAGR^{409}	3	2358.9	787.0	0.014	-	1.8
^{389}HALGHNLGMNHDDDHSSCAGR ^{409}	3	2300.9	767.7	-	0.008	-
^{431}CSRDDLENFLK^{441}	3	1396.7	466.2	0.013	0.010	1.3
^{570}CDNPPPGPGGTHCPGASVEHAVCE^{593}	3	2502.0	834.7	0.003	0.004	0.8
^{585}ASVEHAVCENLPCPK^{598}	2	1710.8	856.0	0.001	0.002	0.5
^{623}LTAVVVDDKPCELYCSPLGK^{642}	3	2264.1	755.4	0.038	0.007	5.4
^{750}IRVVEDKPAHSFLA^{763}	2	1581.9	791.4	0.089	-	WT only

[1]Numerical value correspond to the normalized peak area (PA) ratios: PA (semi-tryptic) / PA (tryptic). Active site mutation (A) and respective wild-type amino acid (E) are underlined. WT, wild-type ADAMTS17; EA, active site mutant ADAMTS17

Supplemental Table 2: Peptides from the MS analysis of ADAMTS17 *O*-fucosylation.

TSR	Sequence	z (n)	[M+nH]^{n} (Da)	[M+nH-Hex]$^{n+}$ (Da)	[M+nH-dHex-Hex]$^{n+}$ (Da)	Calculated mass (Da)
1	^{555}CSRTCGTGARF^{565}	2	791.5	709.9	636.9	636.8
1	^{555}CSRTCGTGARF^{565}	3	528.0	473.7	425.0	424.9
3	^{871}SPCSATCEKGF^{881}	2	776.9	695.3	622.4	622.3
4	^{931}SQCSASCGKGVW^{942}	2	--	--	664.3	663.8
5	^{978}TGDWSTCSSTCGK^{990}	2	820.2	738.8	665.9	666.3

Calculated and observed masses of the glycopeptides in Supplemental Figure 2. All peptides were generated from chymotryptic digests except TSR5, which was generated from a tryptic digest. Three forms of each peptide were detected: fully modified with fucose and glucose ([M+nH]$^{n+}$), the monosaccharide form modified only with fucose ([M+nH-Hex]$^{n+}$) and unmodified form ([M+nH-dHex-Hex]$^{n+}$). Average mass of the peptide was used for theoretical calculations. Note that for TSR 1 two different charge states of the same peptide were observed. Modified residues are underlined.

Supplemental Table 3: Peptides from the LC-MS/MS analysis of the tryptic digest of cross-linked ADAMTS17-PCD.

Sequence Peptide 1	Sequence Peptide 2	Z (n)	[M+nH] (Da)	m/z (Da)	cross-linked / unmodified[1]	
					Band 1	Band 2
DDLENFLK^{441}SK^{443}VSTCLLVTDPR	-	3	2588.3	863.400	Cross-link only	Cross-link only
K^{209}PTWGRPSR[2]	VLTEK^{207}K[2]	5	1939.1	388.628	0.0011	0.00013
GNNQVPGGK^{330}DDPPL	VK^{419}GRNPSDL	4	2531.3	633.581	0.046	n.i.
LK^{441}SK^{443}VSTCL	-	2	1173.7	587.300	0.081	0.08
TEK^{207}KK^{209}PTW[2]	-	2	155.6	578.323	Cross-link only	Cross-link only

[1]Numerical values correspond to peak area (PA) ratios: PA (cross-linked) / PA (unmodified peptides). Band 1 and band 2 refer to Fig. 4C. n.i., not identified. [2]This peptide was only identified in the cross-linked form.

Supplemental Table 4: Peptides used for selective reaction monitoring analysis of NEM- accessible cysteine residues.

Sequence	z (n)	[M+nH] (Da)	M/z (Da)	Prot.	Modification	Ident.
C^{373}VLAEDNGLNLAF	2	1435.7	718.3	CT	IA	+
C^{373}VLAEDNGLNLAF	2	1503.7	752.3	CT	NEM	n.i.
KTKLDPPLDGTEC^{521}GADKW	3	2030.9	677.7	CT	IA	+
KTKLDPPLDGTEC^{521}GADKW	3	2098.9	700.3	CT	NEM	n.i.
SAC^{144}GAAGGLVGLIQL	2	1386.7	693.9	CT	IA	+
SAC^{144}GAAGGLVGLIQL	2	1454.7	727.9	CT	NEM	n.i.
SSC^{406}SRDDLENFLK	3	1570.7	524.2	CT	IA	+
SSC^{406}SRDDLENFLK	3	1638.7	546.9	CT	NEM	n.i.
C^{521}GADKWC^{527}RAGE	3	1309.5	437.2	GC	IA/IA	n.i.
C^{521}GADKWC^{527}RAGE	3	1377.5	459.8	GC	IA/NEM	n.i.
C^{521}GADKWC^{527}RAGE	3	1445.5	482.5	GC	NEM/NEM	n.i.
C^{532}VSKTPIPE	2	1030.5	515.7	GC	IA	n.i.
C^{532}VSKTPIPE	2	1098.5	549.8	GC	NEM	n.i.
GRNPSDLSWSSC^{431}SRDDLE	3	2080.9	694.3	GC	IA	+
GRNPSDLSWSSC^{431}SRDDLE	3	2148.9	716.9	GC	NEM	ni
HLM^{o}C^{500}AGLWC^{508}LVE	2	1504.7	502.2	GC	IA/IA	+
HLM^{o}C^{500}AGLWC^{508}LVE	2	1572.6	524.9	GC	IA/NEM	n.i.
HLM^{o}C^{500}AGLWC^{508}LVE	2	1640.6	547.5	GC	NEM/NEM	n.i.
GRPAELC^{125}FYSGR	3	1412.7	471.6	T	IA	+
GRPAELC^{125}FYSGR	3	1480.6	494.2	T	NEM	n.i.
NMEHLM^{o}C^{495}AGLWC^{500}LVEGDTSC^{508}K	3	2610.9	871.0	T	IA/IA/NEM	n.i.
NM^{o}EHLM^{o}C^{495}AGLWC^{500}LVEGDTSC^{508}K	3	2542.9	848.3	T	IA/IA/IA	+
NPSDLSWSSC^{431}SR	2	1395.6	698.3	T	IA	+
NPSDLSWSSC^{431}SR	2	1463.6	732.3	T	NEM	n.i.
SLESFC^{308}HWQNEEYGGAR	2	2069.9	1035.4	T	IA	+
SLESFC^{308}HWQNEEYGGAR	2	2137.9	1069.4	T	NEM	0.0037
TDFC^{348}VHKDEPC^{355}DTVGIAYLGGVC^{367}SAK	3	2899.3	967.1	T	IA/IA/IA	+
TDFC^{NEM}VHKDEPC^{355}DTVGIAYLGGVC^{367}SAK	3	2967.3	989.8	T	IA/IA/NEM	0.0013
TDFC^{348}VHKDEPC^{NEM}DTVGIAYLGGVC^{367}SAK	3	2967.3	989.8	T	IA/IA/NEM	0.0017
TDFC^{348}VHKDEPC^{355}DTVGIAYLGGVC^{NEM}SAK	3	2967.3	989.8	T	IA/IA/NEM	0.0039
TDFC^{348}VHKDEPC^{355}DTVGIAYLGGVC^{367}SAK	3	3035.2	1012.4	T	IA/NEM/NEM	n.i.
TDFC^{348}VHKDEPC^{355}DTVGIAYLGGVC^{367}SAK	3	3103.2	1035.1	T	NEM/NEM/NEM	n.i.
TKLDPPLDGTEC^{521}GADK	3	1716.8	572.9	T	IA	+
TKLDPPLDGTEC^{521}GADK	3	1784.8	595.6	T	NEM	n.i.
VSTC^{447}LLVTDPR	2	1260.7	630.8	T	IA	+
VSTC^{447}LLVTDPR	2	1328.6	664.8	T	NEM	0.0034
WSLTPSPSAEAQRPEQLC^{201}K	2	2185	1093	T	IA	+
WSLTPSPSAEAQRPEQLC^{201}K	2	2253	1127	T	NEM	0.019

Numerical values correspond to peak area (PA) ratios: PA (NEM modified) / PA (IA modified). CT, chymotrypsin, GC, endoproteinase GluC, T, trypsin, NEM, N-ethylmaleimide, IA, idoacetamide, n.i., not identified.
